# Structural Characterization, Toxicity Assessment and Molecular Modeling of Forced Degradation Products of Siponimod

**DOI:** 10.3390/ijms27083630

**Published:** 2026-04-18

**Authors:** Yajing Liang, Tingting Zhang, Dongfeng Zhang, Bo Jin, Chen Ma

**Affiliations:** Institute of Materia Medica, Chinese Academy of Medical Sciences & Peking Union Medical College, Beijing 100050, China; liangyajing@imm.ac.cn (Y.L.); zhangtingting-87@imm.ac.cn (T.Z.); zdf@imm.ac.cn (D.Z.)

**Keywords:** siponimod, forced degradation, degradation products, in silico toxicity, molecular docking, molecular dynamics

## Abstract

Siponimod, a selective sphingosine 1-phosphate (S1P) receptor modulator, represents a next-generation therapeutic drug for active secondary progressive multiple sclerosis. This study conducted in-depth forced degradation studies of siponimod in solid state subjected to acidic, alkaline, oxidative, photolytic, and thermal conditions, in compliance with ICH guidelines Q1A (R2) and Q3A (R2). An HPLC method was developed to quantify siponimod and separate its degradation products (DPs). The DPs were characterized using LC-HRMS/MS and LC-MS^n^ techniques. Moreover, the toxicological profiles of siponimod and its DPs were evaluated through the in silico tools ProTox 3.0 and ADMETlab 3.0, with molecular docking and dynamics simulations assessing their binding to the S1P1 receptor. Siponimod was stable to light but degraded under acidic, alkaline, oxidative, and thermal stress, producing five products: DP-1 (acidic), DP-2/3 (oxidative), DP-4 (hydrolytic), and DP-5 (thermal). The toxicity prediction suggested that neither siponimod nor its DPs exhibited carcinogenic or mutagenic potential, and the molecular modeling analysis revealed that DP-2 and DP-3 demonstrated favorable binding affinities, with stable dynamic profiles and thermodynamic properties that closely resembled those of siponimod. As far as we know, this is the first study on the structural elucidation of the DPs of siponimod by LC-HRMS/MS and LC-MS^n^.

## 1. Introduction

Siponimod, (*E*)-1-(4-(1-(1-((4-cyclohexyl-3-(trifluoromethyl) benzyl) oxy) imino) ethyl)-2-ethylbenzyl) azetidine-3-carboxylic acid, is an advanced selective modulator of the sphingosine 1-phosphate 1 and 5 (S1P1 and S1P5) receptors and has received regulatory approval in various countries for the treatment of secondary progressive multiple sclerosis (SPMS) [1]. Multiple sclerosis is an autoimmune disorder that affects the central nervous system (CNS) and it is characterized by extensive neurodegeneration, immune cell accumulation in specific regions, and inflammation-driven demyelination [2,3]. It is estimated that multiple sclerosis affects more than 2–3 million people globally, with no current cure available [4]. So far, disease-modifying therapies (DMTs) for relapsing–remitting multiple sclerosis (RRMS) have made substantial progress, but the treatment of SPMS remains challenging [5,6,7]. Siponimod, as the first DMT oral drug approved worldwide for SPMS, can slow the deterioration of disability and cognitive processing abilities, foster myelin regeneration, prevent CNS infiltration and produce an anti-inflammatory response [3,8,9].

Impurities in drug substances are closely associated with the effectiveness and safety of medications [10]. A thorough comprehension of impurities in active pharmaceutical ingredients (APIs) can offer valuable perspectives on the determination of appropriate storage conditions, development of formulations, and selection of manufacturing techniques [11,12,13]. In particular, the International Council for Harmonisation of Technical Requirements for Pharmaceuticals for Human Use (ICH) guidelines specifically recommend that degradation products (DPs) be thoroughly characterized, particularly for those exceeding set thresholds, as they could demonstrate unforeseen toxicity or biological activity [14]. Structural characterization of degradation impurities generated in stress degradation tests and the clarification of their degradation pathways can provide important information regarding the intrinsic characteristics of pharmaceutical molecules, offering novel insights for improving drug safety [15,16].

Anita Kethipalli et al. [17] developed a reversed-phase ultra-high performance liquid chromatography (RP-UPLC) method for the simultaneous quantification of siponimod and ponesimod using isocratic elution. Although a forced degradation test of siponimod was conducted, the purpose was to ensure that the peaks of the DPs did not interfere with that of the APIs in the assay, rather than to specifically establish an analytical method and perform structural elucidation for its DPs. Penchala Reddy Vaka et al. [18] optimized a liquid chromatography-tandem mass spectrometry (LC-MS/MS) method to quantify potential genotoxic impurities during synthesis of siponimod and its formulations, without focusing on the DPs. Additionally, LC-MS/MS-based methods have been developed to assess the pharmacokinetics of siponimod in biological samples such as human [19,20], mice [21], and rats [22]. To the best of our knowledge, no dedicated method has been established for the isolation of siponimod and its DPs and no systematic structural characterization of the DPs of siponimod has been reported. Considering that the DPs formed during prolonged storage, if undetected or uncharacterized, may present toxicological hazards; conducting a systematic forced degradation study is essential to safeguard drug safety and maintain quality.

Herein, we performed a forced degradation study of siponimod in solid state, by subjecting it to various stress conditions, including hydrolytic (acidic and alkaline), oxidative, photolytic and thermal conditions. A liquid chromatography–photodiode array detector (LC-PDA)-based analytical approach was developed to simultaneously separate the DPs and quantify siponimod with validation. The DPs of siponimod were characterized in both positive and negative ion modes using liquid chromatography–high resolution tandem mass spectrometry (LC-HRMS/MS) and liquid chromatography–multi-stage mass spectrometry (LC-MS^n^), and the possible degradation mechanism and pathways of siponimod were proposed. Additionally, the biological safety and molecular interaction characteristics of siponimod and its DPs were investigated through in silico toxicity assessment, molecular docking, and molecular dynamics (MD) simulations.

## 2. Results and Discussion

### 2.1. Optimization of Forced Degradation Conditions

Forced degradation experiments were conducted in accordance with ICH guidelines Q1A (R2) [23] and Q3A (R2) [24]. A series of tests was conducted to select the forced degradation conditions to obtain an appropriate degree of degradation and allow for reliable structural elucidation via LC-MS, including oxidative degradation (3% H_2_O_2_, for 7 h, 24 h, 4 d in the dark), acidic degradation (1 mol/L HCl, for 7 h in the dark; 6 mol/L HCl, for 24 h, 3 d, 4 d in the dark), alkaline degradation (1 mol/L NaOH, for 7 h in the dark; 6 mol/L NaOH, for 24 h, 3 d, 4 d in the dark), thermal degradation (60 °C, for 4 d; 80 °C, for 24 h, 48 h) and photolytic degradation (5000 lux, for 7 h, 4 d, 10 d) tests. The final conditions are shown in Table 1.

### 2.2. LC-PDA and LC-MS Method Development

The optimized LC methods involved adjustments to the column types, mobile phase, column temperature, and elution conditions. Initially, the Kromasil Eternity C18 column (Nouryon, Göteborg, Sweden) (4.6 × 250 mm, 5 µm) and alkali-resistant ChromCore BR C18 column (NanoChrom Technologies Co., Ltd., Suzhou, China) (4.6 × 150 mm, 3 µm) were selected to separate siponimod and its DPs. The investigation of the mobile phase included a combination of deionized water, 0.1% formic acid aqueous solution, and 10 mmol/L ammonium formate buffer solutions (pH values adjusted to 8.0, 8.5, 9.0 and 9.5, respectively), in varying ratios with acetonitrile. The experimental results revealed that the ammonium formate buffer notably increased the symmetry of the chromatographic peaks, and that the retention time and resolution of siponimod and its DPs were significantly influenced by the pH of the mobile phase. As the pH increased, the separation of siponimod and closely eluting DPs improved. The optimal resolution and peak symmetry were achieved at pH 9.5. As a result, the alkali-resistant ChromCore BR C18 column with the mobile phase consisting of acetonitrile and ammonium formate buffer (pH 9.5) was used for the separation of siponimod and its DPs. Moreover, the impact of the column temperature at 30 °C and 40 °C was examined, and good resolution of DP-2 and DP-3 in H_2_O_2_ sample solution was observed at 30 °C. Considering the easy elution of DP-1, a high proportion of the aqueous phase (90%) was used as the initial ratio for gradient elution to ensure effective separation of DP-1 from the solvent peak.

To achieve more precise detection of DPs, both positive and negative ion mode full scans for electrospray ionization (ESI) were employed to acquire data. For HRMS/MS, the mass spectrometric parameters, particularly the collision energy, were optimized to achieve high signal intensity and sensitivity. For MS^n^, the declustering potential and collision energy were optimized on the basis of the fragmentation pattern of DPs to increase the ion fragmentation efficiency.

### 2.3. Stress Decomposition Behavior

**Figure 1:**
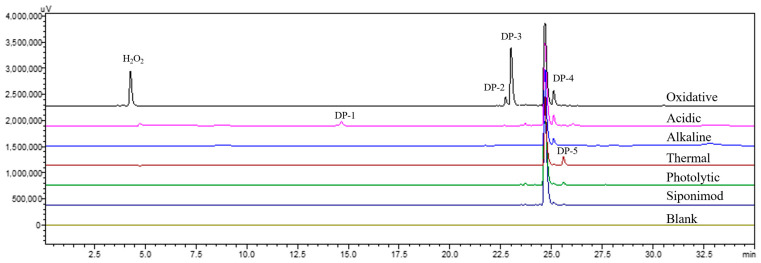
Chromatograms of siponimod under different degradation conditions at the wavelength of 254 nm.

Figure 1 depicts the overlapped chromatograms obtained from the forced degradation samples of siponimod analyzed by LC-PDA. A total of five DPs were detected, with the peaks numbered according to their elution sequence. The percentages of DPs determined by the area normalization method under different conditions are summarized in Table 1. The results indicated that siponimod experienced degradation under acidic hydrolysis (DP-1, DP-4), alkaline hydrolysis (DP-4), oxidative (DP-2, DP-3, and DP-4), and thermal (DP-5) conditions, while no noticeable degradation occurred under photolytic conditions. The findings offered valuable perspectives on the practical stability of siponimod, guiding decisions regarding optimal packaging, storage, and formulation strategies.

### 2.4. Validation Results of the LC-PDA Method

The method validation experiments were performed in compliance with the ICH guidelines Q2 (R2) [25], and all evaluated parameters satisfied the stipulated criteria (see Section 3.4.7). Regarding specificity, HPLC separation of the forced degradation samples showed well-defined baseline separation and sufficient chromatographic resolution. As illustrated in Figure 1, the resolutions of DP-4 and siponimod in oxidative, acidic, and alkaline stress test solutions were found to be 1.8, 1.8, and 1.9, respectively. PDA-based evaluation of peak purity verified the consistency of the peaks, with values for siponimod (0.9996), DP-1 (0.9759), DP-2 (0.9984), DP-3 (0.9976), DP-4 (0.9985), and DP-5 (0.9900) surpassing the predefined limit. Detailed peak purity plots are available in the Supplementary Materials (Figure S1). No significant interference from either the blank or test solutions was observed with siponimod detection, thereby highlighting the method’s exceptional specificity. The limits of detection and quantitation (LOD and LOQ) were determined to be 0.5 µg/mL and 1.0 µg/mL, respectively. Siponimod demonstrated excellent linearity across a concentration range of 1.062–265.5 µg/mL, with a linear regression equation of y = 32,429x − 16,158 (r = 0.9999). Residual analysis was additionally performed to evaluate the appropriateness of the linear regression model. The plot of residuals against concentration (Figure S2 in the Supplementary Materials) revealed no discernible systematic patterns, suggesting an adequate fit to the model. The solution maintained stable for a duration of 72 h, with a peak area ratio (A_72h_/A_0h_) of 1.0. The intra-day relative standard deviation (RSD) of the peak area of siponimod was 0.2%, and the inter-day RSD was 0.5%. Furthermore, the recoveries for the six samples were 99.8%, 98.4%, 99.4%, 100.6%, 97.9%, and 98.6%, resulting in an average recovery of 99.1% and an RSD of 1.0%, all of which fall within the acceptable thresholds specified by the ICH guidelines. The method’s robustness was assessed by systematically altering key chromatographic parameters. In particular, the separation between siponimod and the adjacent impurity consistently maintained a resolution greater than 1.5 across the flow rate range of 0.4–0.6 mL/min, the mobile phase pH between 9.2 and 9.8, and the column temperature spanning from 25 to 30 °C. Collectively, these observations indicated that minor deviations in flow rate, pH, or temperature did not adversely affect the chromatographic separation, thereby confirming the method’s robustness.

System suitability tests were conducted prior to the analytical run to verify optimal chromatographic performance. For the standard solution, siponimod showed a theoretical plate count of 60,035. The tailing factor was measured at 1.2, and the repeatability of replicate injections was 0.2%, well below the maximum allowed value of ≤2%. Moreover, in the alkaline hydrolysis sample solution, the resolution between siponimod and DP-4 was 1.9, meeting the criterion of ≥1.5. Collectively, these system suitability test outcomes indicated that the method was appropriate for analysis.

### 2.5. Characterization of Siponimod and Its DPs

The structures of siponimod and its DPs were extensively elucidated using LC-HRMS/MS and LC-MS^n^ in both positive and negative ion modes. It is interesting to find that the data obtained in both ion modes offered complementary insights into the structural characterization of the DPs. For the majority of DPs (DP-2, -3, -4, and -5), the positive ion mode results aligned with those of siponimod, generating identical fragment ions at *m*/*z* 416, *m*/*z* 348, *m*/*z* 241, *m*/*z* 178, and *m*/*z* 159. In contrast, negative ion mode provided more detailed structural information, which facilitated the differentiation of distinct moieties in the DPs. In view of this, siponimod and DP-1 were characterized in positive and negative modes, and the remaining four DPs were analyzed exclusively in negative ion mode. Indraw 7.1 was employed to generate the corresponding molecular structures.

According to the HRMS/MS spectra data, the observed mass, the calculated exact mass, and the elemental compositions corresponding to the proposed molecular formulas of the parent and product ions are detailed in Table 2 (positive ion mode) and Table 3 (negative ion mode).

#### 2.5.1. Siponimod

**Figure 2:**
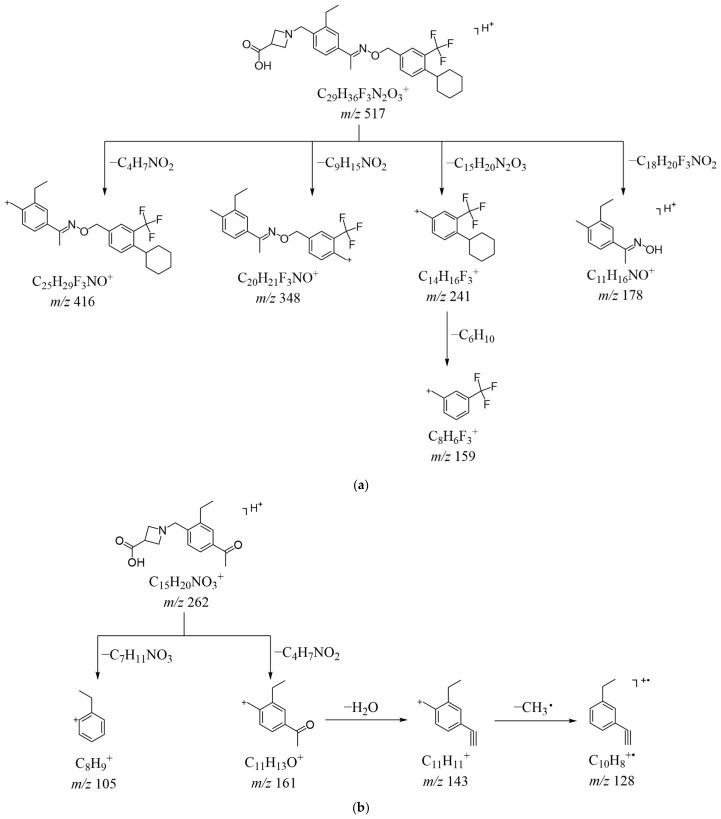
Potential fragmentation pathways in ESI (+): (**a**) siponimod; (**b**) DP-1.

The protonated molecular ion of siponimod was observed at *m*/*z* 517.2665 (C_29_H_36_F_3_N_2_O_3_^+^) (Figure S3 in the Supplementary Materials) and the HRMS/MS spectrum in positive ion mode is shown in Figure S4. The parent ion fragmented between the oxygen group and the benzyl group, and [M + H]^+^ lost C_15_H_20_N_2_O_3_ to form a product ion at *m*/*z* 241, followed by the loss of a saturated six-membered ring (C_6_H_10_) to yield a base peak at *m*/*z* 159. The loss of C_4_H_7_NO_2_, C_9_H_15_NO_2_, and C_18_H_20_F_3_NO_2_ from [M + H]^+^ yielded product ions at *m*/*z* 416, *m*/*z* 348, and *m*/*z* 178, respectively, which exhibited cleavage sites at the carbon-nitrogen and carbon-oxygen single bonds, as well as at the benzylic carbon connecting the benzene ring to the six-membered ring. The proposed fragment ions can be supported by MS^n^ (Figure S5 in the Supplementary Materials), and the possible fragmentation pathway of siponimod in positive ion mode is depicted in Figure 2a.

**Figure 3:**
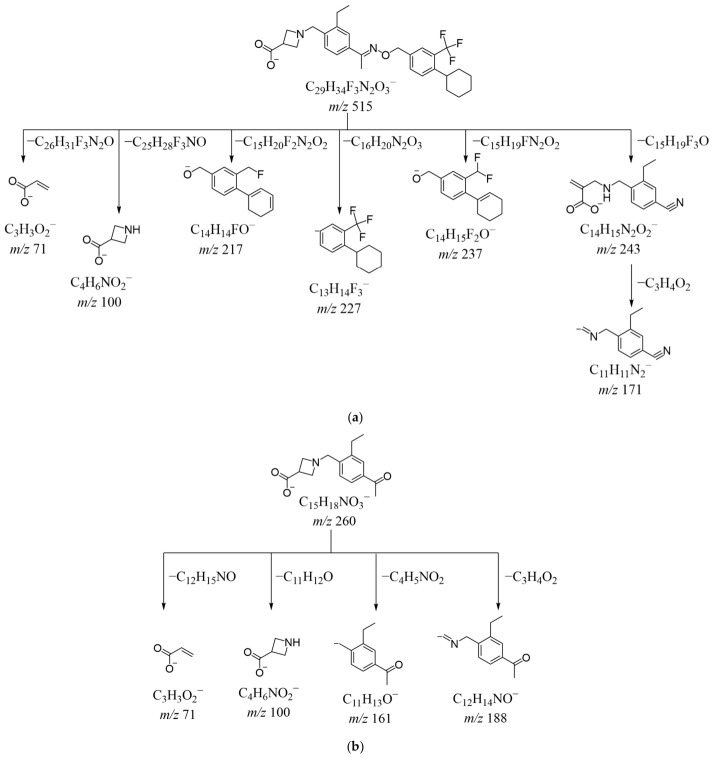

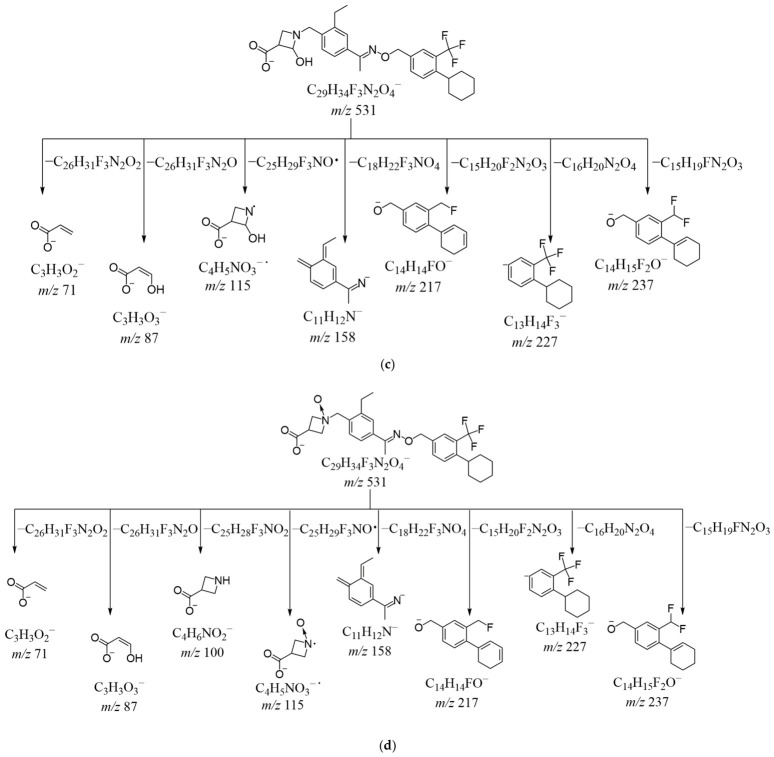

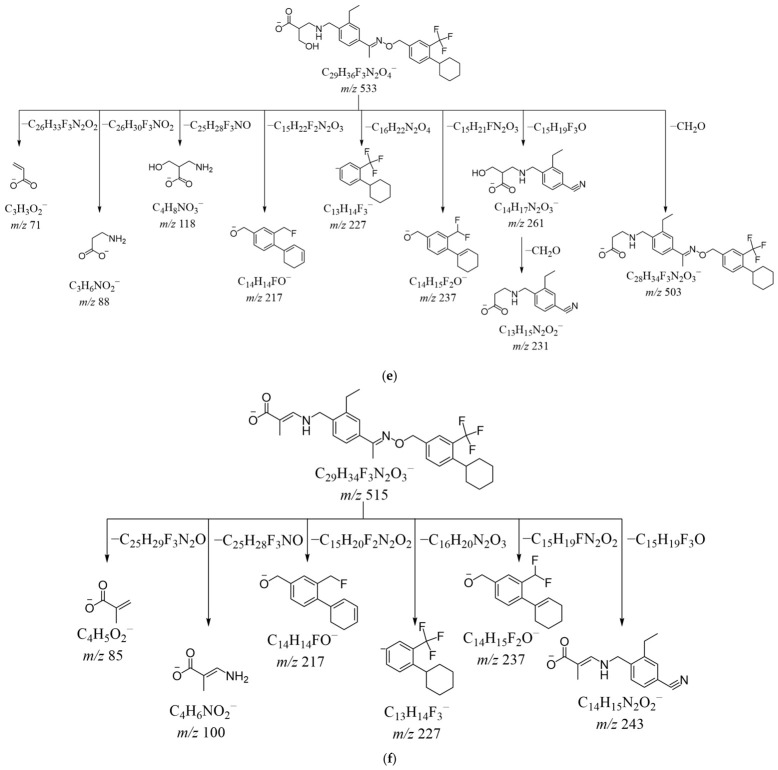
Potential fragmentation pathways in ESI (−): (**a**) siponimod; (**b**) DP-1; (**c**) DP-2; (**d**) DP-3; (**e**) DP-4; (**f**) DP-5.

The [M − H]^−^ ion of siponimod was observed at *m*/*z* 515.2518 (C_29_H_34_F_3_N_2_O_3_^−^) (Figure S6 in the Supplementary Materials). The HRMS/MS spectrum of siponimod in negative ion mode is shown in Figure S7. The daughter ions at *m*/*z* 237 and *m*/*z* 217 came from [M − H]^−^ by the loss of C_15_H_19_FN_2_O_2_ and C_15_H_20_F_2_N_2_O_2_, respectively, which were produced by breaking the nitrogen–oxygen single bond of the parent ion and were accompanied by the loss of HF. The loss of C_16_H_20_N_2_O_3_ from [M − H]^−^ generated the fragment ion at *m*/*z* 227. The [M − H]^−^ ion lost C_15_H_19_F_3_O to form the most abundant ion at *m*/*z* 243, followed by the elimination of C_3_H_4_O_2_ to form the fragment ion at *m*/*z* 171. The nitrogen-containing four-membered heterocycle in the structure of siponimod could be ring-opened to form double bonds, which was consistent with the azetidine cleavage pattern described in the literature by Ana Čikoš et al. [26] and R. G. Kostyanovsky et al. [27]. The carbon-nitrogen single bond in siponimod broke, and the loss of C_25_H_28_F_3_NO and C_26_H_31_F_3_N_2_O from [M − H]^−^ gave rise to daughter ions at *m*/*z* 100 and *m*/*z* 71, respectively, providing additional structural insight into the 1-azetidine-3-carboxylic acid group, which was not detected in positive ion mode. The MS^n^ spectrum of siponimod in negative ion mode is shown in Figure S8. Considering the above information, Figure 3a illustrates the probable fragmentation pathway of siponimod in negative ion mode.

#### 2.5.2. DP-1

The [M + H]^+^ ion of DP-1 was observed at *m*/*z* 262.1429 (C_15_H_20_NO_3_^+^) (Figure S9), a decrease of 255 Da compared with the [M + H]^+^ ion of siponimod, which implied that the imino group in siponimod could have undergone conversion to a carbonyl group, thereby resulting in the formation of DP-1. The HRMS/MS spectrum of DP-1 in positive ion mode is shown in Figure S10. The ion at *m*/*z* 161 was obtained by the loss of C_4_H_7_NO_2_ from [M + H]^+^ of DP-1, which was the same as the fragment lost from siponimod during the transition from *m*/*z* 517 to *m*/*z* 416. The ion at *m*/*z* 161 dehydrated to form the fragment ion at *m*/*z* 143, followed by the loss of CH_3_• to give the most abundant ion at *m*/*z* 128. The [M + H]^+^ ion lost C_7_H_11_NO_3_ to yield *m*/*z* 105. The proposed fragment ions can be corroborated by MS^n^ data (Figure S11).

The [M − H]^−^ ion of DP-1 was observed at *m*/*z* 260.1290 (C_15_H_18_NO_3_^−^) (Figure S12). The HRMS/MS spectrum of DP-1 in negative ion mode is shown in Figure S13. The [M − H]^−^ ion lost C_11_H_12_O and C_12_H_15_NO to obtain the ion at *m*/*z* 100 and the most abundant ion at *m*/*z* 71, respectively, and these fragment ions were consistent with those observed in siponimod. The loss of C_4_H_5_NO_2_ and C_3_H_4_O_2_ from [M − H]^−^ gave the daughter ions at *m*/*z* 161 and *m*/*z* 188. Notably, the fragment loss pattern of [M − H]^−^ to the ion at *m*/*z* 188 in DP-1 was identical to that observed during the transition from *m*/*z* 243 to *m*/*z* 171 in siponimod. Based on the above information, DP-1 was possibly 1-(4-acetyl-2-ethylbenzyl) azetidine-3-carboxylic acid. The structure of DP-1 proposed in this study aligned with that of the metabolite M1 in urine after the administration of siponimod [28]. Combined with the primary mass spectra, HRMS/MS spectra and MS^n^ spectra, the cleavage pathways of DP-1 are shown in Figure 2b (positive) and Figure 3b (negative).

#### 2.5.3. DP-2

The [M − H]^−^ ion of DP-2 was observed at *m*/*z* 531.2477 (C_29_H_34_F_3_N_2_O_4_^−^) (Figure S14), which was 16 Da (O) greater than the [M − H]^−^ ion of siponimod. The HRMS/MS spectrum of DP-2 in negative ion mode is shown in Figure S15. With the same cleavage mechanism as siponimod, [M − H]^−^ lost C_15_H_19_FN_2_O_3_, C_16_H_20_N_2_O_4_, C_15_H_20_F_2_N_2_O_3_ and C_26_H_31_F_3_N_2_O_2_ to give *m*/*z* 237, *m*/*z* 227, *m*/*z* 217, and the most abundant ion at *m*/*z* 71, respectively, corresponding to the fragment ions of siponimod. The [M − H]^−^ ion of DP-2 lost C_18_H_22_F_3_NO_4_ and C_26_H_31_F_3_N_2_O to give ions at *m*/*z* 158 and *m*/*z* 87. The [M − H]^−^ ion and the ion at *m*/*z* 87 were 16 Da greater than the [M − H]^−^ ion and the ion at *m*/*z* 71 of siponimod, indicating the presence of a hydroxyl group on the nitrogen-containing four-membered ring. Similarly, the odd-electron ion at *m*/*z* 115 was 15 Da greater than the ion at *m*/*z* 100 of siponimod, providing additional information regarding the position of the hydroxyl group. To summarize, the structure of DP-2 was presumed to involve the addition of a hydroxyl group to the nitrogen-containing four-membered ring of siponimod. The cleavage pathway is presented in Figure 3c.

#### 2.5.4. DP-3

The [M − H]^−^ ion of DP-3 was observed at *m*/*z* 531.2473 (C_29_H_34_F_3_N_2_O_4_^−^) (Figure S16), an increase of 16 Da (O) compared with that of siponimod, which should be isomeric with that of DP-2. According to the HRMS/MS data in negative ion mode in Figure S17, the daughter ions obtained at *m*/*z* 237, *m*/*z* 227, *m*/*z* 217, *m*/*z* 158, *m*/*z* 115, *m*/*z* 87, and *m*/*z* 71 were identical to those of DP-2, with the ion at *m*/*z* 71 serving as the base peak. In contrast to that of DP-2, the HRMS/MS spectrum of DP-3 showed a peak at *m*/*z* 100, corresponding to that observed in siponimod. It was reasonable to speculate that DP-3 was a nitrogen-oxide derivative of siponimod, where the [M − H]^−^ ion of DP-3 could undergo cleavage at the covalent bond between the nitrogen and oxygen atoms [29], as well as at the carbon-nitrogen bond analogous to siponimod, in contrast to DP-2, which lost a hydroxyl group and dehydrated to form a double bond [30], ultimately generating the ion at *m*/*z* 100. The potential cleavage pathway of DP-3 is illustrated in Figure 3d.

#### 2.5.5. DP-4

The [M − H]^−^ ion of DP-4 was observed at *m*/*z* 533.2634 (C_29_H_36_F_3_N_2_O_4_^−^) (Figure S18), which was an increase of 18 Da (H_2_O) greater than that of siponimod. The HRMS/MS spectrum of DP-4 in negative ion mode is shown in Figure S19. The ions at *m*/*z* 237, *m*/*z* 227, *m*/*z* 217 and *m*/*z* 71 were all the same as the fragments produced by siponimod. The [M − H]^−^ ion, and the ions at *m*/*z* 118 and *m*/*z* 261 were 18 Da greater than the [M − H]^−^ ion, and the fragment ions at *m*/*z* 100 and *m*/*z* 243 produced by siponimod, suggesting ring-opening hydrolysis of azetidine in siponimod. The [M − H]^−^ ion lost CH_2_O to form the ion at *m*/*z* 503, and similarly, the ion at *m*/*z* 261 lost CH_2_O, resulting in the formation of the ion at *m*/*z* 231. Additionally, the loss of C_26_H_30_F_3_NO_2_ from [M − H]^−^ produced the ion at *m*/*z* 88 (C_3_H_6_NO_2_^−^). These fragmentation patterns implied that DP-4 likely contained a structural unit of 3-amino-2-(hydroxymethyl) propanoic acid. The MS^n^ spectrum of DP-4 in negative ion mode is shown in Figure S20. Based on the above information, the proposed cleavage pathway of DP-4 is depicted in Figure 3e.

#### 2.5.6. DP-5

The primary mass spectrum in negative ion mode revealed that the [M − H]^−^ ion of DP-5 was at *m*/*z* 515.2521 (C_29_H_34_F_3_N_2_O_3_^−^) (Figure S21), having the same molecular weight as siponimod; therefore, it was inferred to be an isomer of siponimod. The HRMS/MS spectrum in negative ion mode is shown in Figure S22. Due to the presence of fragment ions at *m*/*z* 243, *m*/*z* 237, *m*/*z* 227, *m*/*z* 217, and *m*/*z* 100, the mass spectral cleavage pattern of DP-5 was basically consistent with that of siponimod. Unlike the fragment ion at *m*/*z* 71 produced by siponimod, the ion at *m*/*z* 85 was observed in DP-5, indicating the presence of methacrylic acid. Combining the above fragmentation information, it was reasonable to deduce that the structure of DP-5 may have changed from the 1-azetidine-3-carboxylic acid moiety of siponimod to the 3-amino-2-methacrylic acid moiety. The hypothesized cleavage pathway of DP-5 is presented in Figure 3f.

### 2.6. Postulated Degradation Pathways of Siponimod

**Figure 4:**
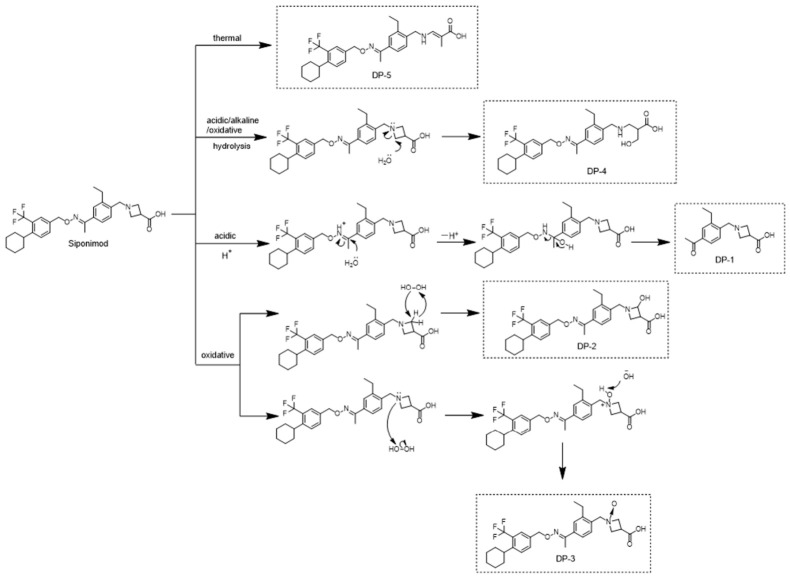
Postulated degradation mechanism of siponimod.

Figure 4 displays the plausible degradation pathways of siponimod under different degradation conditions. The imine group in siponimod was sensitive to acidic conditions. When exposed to hydrochloric acid, H_2_O acted as a nucleophile, attacking the imine group and removing the amine fragment, thereby forming a carbonyl group and resulting in the formation of DP-1. In the presence of H_2_O_2_, hydroxyl (-OH) carried out a nucleophilic attack on the azetidinium, leading to the formation of DP-2. Additionally, nitrogen lone-pair electrons within the tetradentate ring interacted with the oxygen atom of H_2_O_2_, forming a covalent ligand bond and producing the nitrogen oxide impurity DP-3. Under acidic, basic, and oxidative conditions, siponimod underwent hydrolysis of the nitrogen-containing heterocyclic ring, followed by electrophilic addition with water to yield DP-4. DP-5, an isomer of siponimod bearing a methacrylic acid fragment, was generated through thermal processes.

### 2.7. In Silico Toxicity Prediction

This study focused on evaluating the toxicity profiles of siponimod and its five DPs through computational approaches. Predicting toxicity at early stages is essential in drug development, as it enables identification of potential chemical hazards before undertaking resource-intensive in vitro or in vivo studies [31]. To this end, two complementary online platforms, ProTox 3.0 [32] and ADMETlab 3.0 [33], were applied to estimate critical toxicological endpoints, including mutagenic and carcinogenic potential.

ProTox 3.0 was employed due to its specialized capability in forecasting mutagenic and carcinogenic potentials, utilizing an extensive repository of established mutagens and carcinogens combined with machine learning-driven similarity scoring [32]. This approach permits a focused assessment of the genetic and oncogenic risks associated with the DPs. In parallel, ADMETlab 3.0 facilitated a more comprehensive evaluation of toxicity, covering general toxicological profiles, pharmacokinetic properties (ADME), and overall safety parameters [33]. The combined application of these two platforms enabled a thorough and systematic investigation, allowing the identification of potentially high-risk impurities and providing a deeper understanding of the overall hazard profile of siponimod and its DPs.

Table 4 summarizes the prediction results for carcinogenicity and mutagenicity, showing consistent outcomes across both platforms, along with probability scores (range: 0–1) that indicate the confidence level of each prediction, with higher scores representing greater confidence. The findings revealed that siponimod and its five DPs had minimal carcinogenicity, with all the carcinogenicity predictions being negative. Furthermore, both siponimod and the DPs exhibited low mutagenic potential, with negative predictions for mutagenicity. This suggested that all compounds were predicted to be devoid of structural motifs linked to DNA reactivity or mutagenic potential, implying a low likelihood of inducing genetic mutations.

Computational analysis indicated that siponimod and its DPs largely possessed a favorable safety profile. Nevertheless, the lack of structural alerts could not ensure complete safety, since adverse effects may emerge via non-genotoxic pathways, such as receptor-mediated interactions, oxidative stress, or mitochondrial impairment [34]. While such in silico methods were instrumental for initial toxicity assessment, they cannot replace empirical verification, as their accuracy was contingent upon the comprehensiveness and quality of the databases and algorithms employed [35]. Consequently, subsequent investigations should validate these outcomes using both in vitro and in vivo experimental models. Additionally, this research primarily addressed carcinogenic and mutagenic potentials, leaving other pivotal toxicity endpoints—such as neurotoxicity, immunotoxicity, and developmental toxicity—largely unexplored. Incorporating these dimensions would enable a more thorough appraisal of safety.

### 2.8. Molecular Modeling Studies

The molecular docking strategy provides an in-depth insight into interactions between proteins and between receptors and ligands. This approach allows for the assessment of binding affinity in physiologically relevant conditions and delivers essential physicochemical information regarding intermolecular interactions among proteins, enzymes, and ligands [36,37]. Molecular docking simulations were performed to investigate interactions between siponimod, its DPs, and the S1P1 receptor, represented by PDB ID: 7EVY, retrieved from the Protein Data Bank. This structure was selected based on three criteria: (1) biological relevance: it corresponds to the human S1P1 receptor, the direct therapeutic target of siponimod [38]; (2) structural quality: resolved at 2.98 Å, providing sufficient accuracy for reliable docking, comparable to other S1P1 structures (e.g., PDB ID: 7EO4, 2.86 Å) [39]; (3) literature support: 7EVY has been widely used in S1P1-targeted drug discovery and structure–activity relationship (SAR) studies, including ligand-binding characterization [40,41].

**Figure 5:**
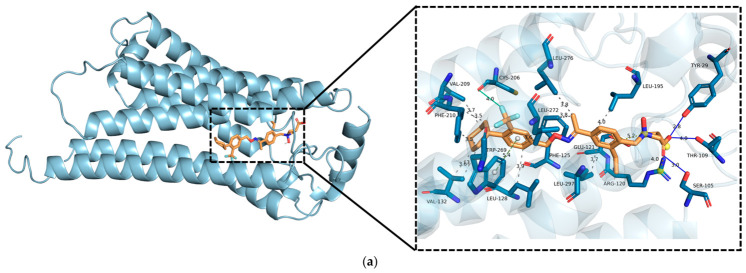

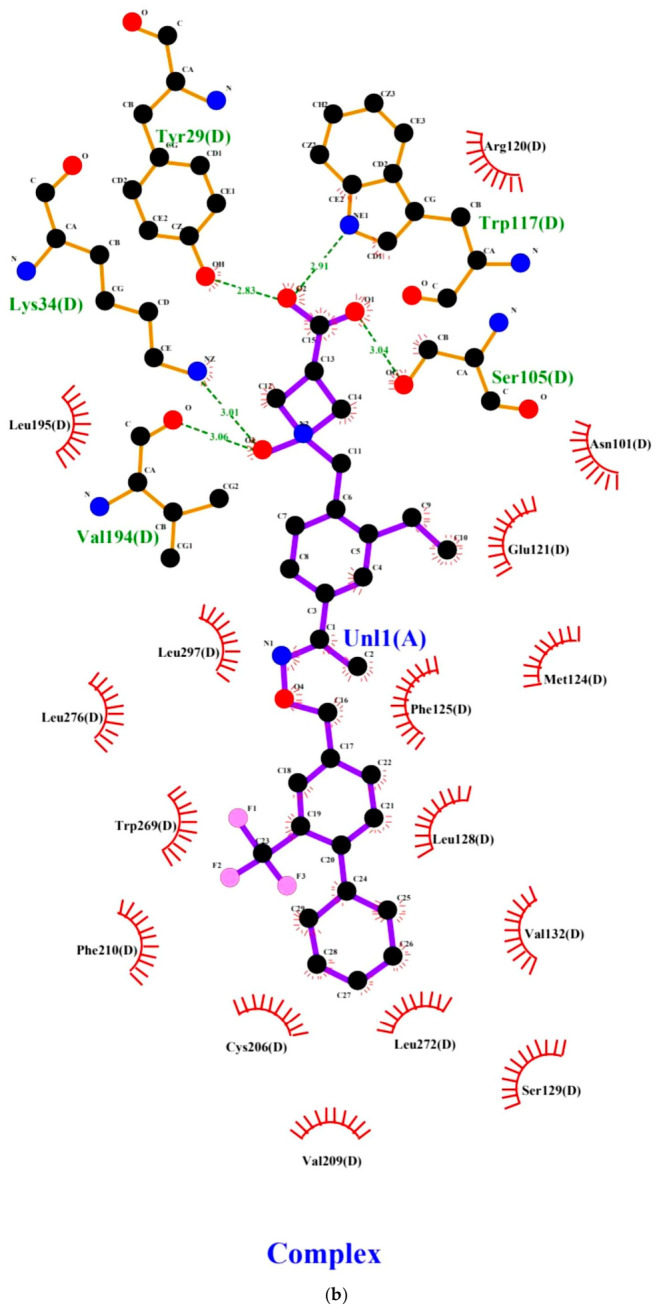
Docking interaction of DP-3 3D (**a**) and 2D (**b**) diagrams. The central purple lines represent the ligand molecule, and the green dashed lines denote hydrogen-bond interactions. The connected brown structure corresponds to the amino acid participating in these interactions, and the red "lashes" adjacent to the receptor indicate the names of the surrounding amino acids.

To verify the reliability of the docking results, the root-mean-square deviation (RMSD) between the S1P1 receptor’s crystal structure and the docked pose of siponimod was determined, with the RMSD calculated to be 2 Å. This result was within the acceptable limits for docking validation, implying that the docking method generated a fairly accurate pose in relation to the experimentally derived crystal structure. Surface maps and corresponding ligand-receptor interactions are shown in Figure 5 (DP-3) and Figures S23–S27, while the affinity of the ligands is presented in Table 5. It was observed that the binding energies of DP-2 and DP-3 were slightly lower than siponimod, which implies that modifications in their structures could potentially enhance their compatibility with the S1P1 receptor. Conversely, DP-1 demonstrated a significantly less favorable binding profile. The comparative analysis of key amino acids in the active site of the S1P1 receptor that interact with siponimod and its DPs is detailed in Table 6. Based on the results, siponimod demonstrated a pronounced binding affinity, which was maintained through a combination of hydrophobic interactions, hydrogen bonds, and pivotal ionic contacts with Lys34, Arg120, and Glu121. An additional halogen bond involving Cys206 further reinforced the interaction. This network collectively established the canonical binding conformation of the parent compound. Among the DPs, DP-3 exhibited the highest binding strength, preserving the essential hydrogen bond triad (Tyr29, Ser105, Thr109), critical salt bridges (Arg120, Glu121), a halogen linkage with Cys206, and an extra π–π stacking interaction with Trp269. These synergistic interactions may account for its enhanced binding energy relative to siponimod. DP-2’s improved affinity may be primarily attributed to extensive hydrophobic contacts and a distinct halogen bond with Phe125, despite alterations in the hydrogen bonding pattern (Ala293, Glu294). In contrast, DP-1 displayed the weakest binding, which may reflect its inability to interact with key polar residues and the absence of stabilizing halogen or π–π stacking interactions, relying instead on limited hydrophobic contacts and a single hydrogen bond with Ser129.

To comprehensively assess the potential activity and stability of DPs, 100 ns MD simulations were conducted for each ligand–S1P1 receptor complex. Key structural and dynamic parameters, including RMSD, root-mean-square fluctuation (RMSF), solvent-accessible surface area (SASA), hydrogen bond count, and radius of gyration (Rg), were examined in conjunction with 3D free energy landscapes. This method, in conjunction with molecular docking, enables the observation of time-dependent conformational alterations and the molecular interaction dynamics between the ligand and receptor at the atomic scale [36].

**Figure 6:**
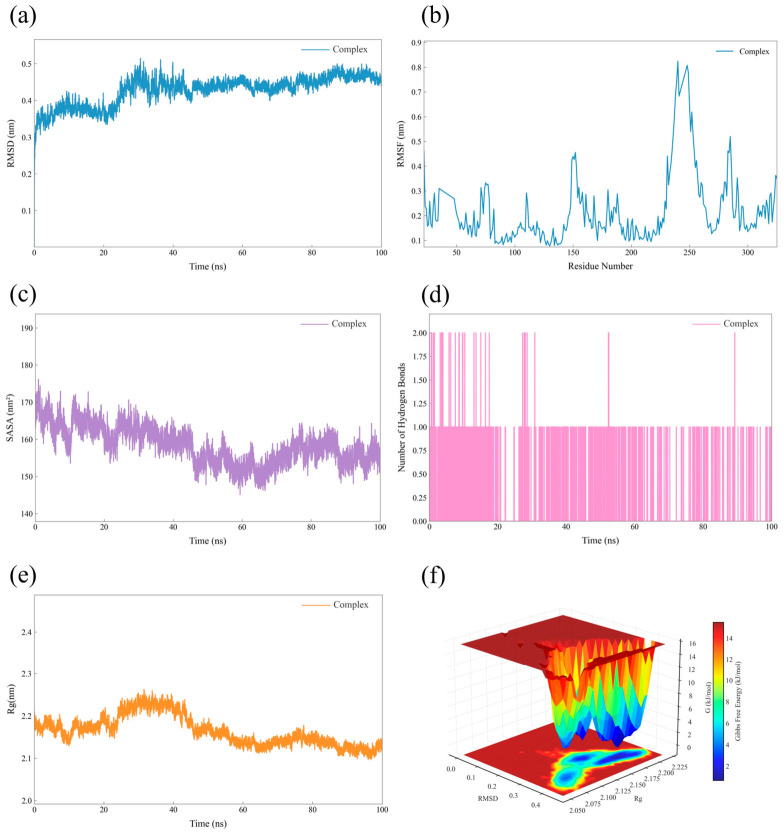
Examination of MD simulations data of DP-3: (**a**) RMSD of the complex; (**b**) Variation in RMSF of the complex; (**c**) SASA of the complex; (**d**) Count of hydrogen bonds within the complex; (**e**) Fluctuations in Rg of the complex; (**f**) Three-dimensional free energy profile.

The MD simulations results are presented in Figure 6 (DP-3) and Figures S28–S32. Throughout the simulation period, all complexes involving siponimod and DPs bound to the S1P1 receptor maintained conformational stability, with no significant structural deviations observed. The RMSD values remained under 0.8 nm without noticeable drift, demonstrating the preservation of structural integrity. RMSF analysis revealed only minor variations across the majority of residues, indicating that ligand binding imposed rigidity on the protein backbone. Additionally, the SASA and Rg profiles remained relatively constant, reflecting the maintenance of compact structures and stable binding interfaces. The hydrogen bond counts between ligands and the receptor showed no significant temporal variation, suggesting that the crucial polar interactions identified during docking were sustained rather than transient. Furthermore, the three-dimensional free energy landscapes of all complexes displayed a single, distinct minimum (approximately 0 kJ/mol), indicating convergence toward energetically favorable and stable conformations.

Based on the results of the molecular docking and MD simulations, DP-2 and DP-3 exhibited favorable binding affinities and retained stable conformational dynamics, comparable to those of siponimod. These results offered computational validation that DP-2 and DP-3 formed stable, dynamic complexes with S1P1, thus reinforcing their potential as bioactive compounds.

## 3. Materials and Methods

### 3.1. Chemicals and Reagents

Siponimod (BAF312) was purchased from MedChemExpress (Monmouth Junction, NJ, USA). Acetonitrile (HPLC grade) was obtained from Honeywell International Inc. (Charlotte, NC, USA). The 10% ammonia and ammonium formate of HPLC grade were purchased from ANPEL Laboratory Technologies Inc. (Shanghai, China). Guaranteed reagent-grade hydrochloric acid was purchased from Beijing Tong Guang Fine Chemicals Company (Beijing, China). Sodium hydroxide (analytical grade) was purchased from Fuchen Chemical Reagent Company (Tianjin, China). Hydrogen peroxide (H_2_O_2_, 3%, analytical reagent grade) was obtained from Sigma-Aldrich Corporation (St. Louis, MO, USA). The deionized water was generated using a water purification system of Milli-Q Reference (Billerica, MA, USA).

### 3.2. Instruments and Methods

#### 3.2.1. LC-PDA

Chromatographic separations were conducted using a Shimadzu LC-20AT system (Kyoto, Japan) fitted with a ChromCore BR C18 column (4.6 × 150 mm, 3 µm, NanoChrom Technologies Co., Ltd.) maintained at 30 °C and a PDA detector scanning from 190 to 400 nm. The mobile phase was composed of ammonium formate buffer (10 mmol/L, pH adjusted to 9.5) (Solvent A) and acetonitrile (Solvent B), and was applied in gradient elution mode at a flow rate of 0.5 mL/min. The gradient solvent program was configured as follows: 0–10 min, 90–45% A; 10–13 min, 45–15% A; 13–23 min, 15% A; 23–25 min, 15–90% A; 25–35 min, 90% A. The detection wavelength was set to 254 nm, and the injection volume was 10 µL.

#### 3.2.2. LC-HRMS/MS

The HRMS/MS studies of siponimod and its DPs were carried out using a Q Exactive orbitrap high-resolution mass spectrometer coupled with a Dionex Ultimate 3000 chromatography system (Thermo Fisher Scientific Inc., Waltham, MA, USA). The chromatographic conditions were identical to those described in Section 3.2.1.

Mass spectrometry scans were conducted in both positive and negative ion modes. The parameters for ESI were set as follows: Spray voltage, 3200 V; Capillary temperature, 320 °C; Sheath gas, 35 arb; Aux gas, 10 arb; Resolution, 17,500; AGC target, 5 × 10^4^; Isolation window, 3.0 *m*/*z*; Scanning range, *m*/*z* 100–1500; Collision energy, (+) 35 V, (−) 35 V. System operation, along with data acquisition and analysis, was executed using Xcalibur 4.2 software.

#### 3.2.3. LC-MS^n^

An AB SCIEX QTRAP 5500 mass spectrometer (Foster City, CA, USA) was combined with a Shimadzu UFLC-20ADXR system for sample analysis in both positive and negative ESI modes. The LC conditions were consistent with those described in Section 3.2.1.

The MS^n^ scanning conditions were optimized in the following manner: IonSpray Voltage (IS), (+) 5500 V or (−) 4500 V; Collision gas, medium; Source temperature (TEM), (+) 500 °C or (−) 450 °C; Curtain gas (CUR), 35 psi; Entrance potential (EP), (+) 10 V or (−) 10 V; Declustering potential (DP), (+) 20 V or (−) 20 V for DP-1, (−) 40 V for the rest DPs; Collision energy (CE), (+) 30 V or (−) 30 V for DP-1, (−) 40 V for DP-2 and DP-3, (−) 45 V for DP-4 and DP-5; Collision cell exit potential (CXP), (+) 10 V or (−) 10 V; Nebulizer source gas 1, 60 psi; Turbo source gas 2, 60 psi. The Analyst 1.6 software was employed for device operation, data collection and processing.

### 3.3. Forced Degradation Studies of Siponimod in Solid State

An accurate amount of 1 mg of siponimod powder was weighed. After the forced degradation experiment was completed, the powder was dissolved and diluted in acetonitrile to achieve a final concentration of 0.5 mg/mL. The thermal stress testing was prepared by placing 1 mg of siponimod at 80 °C in a hot air oven (Hengke, DZF-6020, Shanghai, China) for 24 h. The light degradation was obtained by exposing 1 mg of sample powder to a total irradiance of no less than 1.2 × 10^6^ lux·hr in an illumination incubator (Donglian, HPG-280B, Harbin, China). Following these treatments, both were dissolved and diluted to 0.5 mg/mL with 2 mL of acetonitrile. Oxidative stress was induced by treating 1 mg of siponimod powder with 0.2 mL of 3% H_2_O_2_ solution for 24 h in the dark, followed by dissolution in 1.8 mL of acetonitrile. The acid stress testing was formulated by incubating 1 mg of siponimod with 0.1 mL of 6 mol/L HCl solution for 3 days in the dark, followed by neutralization with 0.1 mL of 6 mol/L NaOH and dissolution in 1.8 mL of acetonitrile. For the base stress condition, 1 mg of siponimod powder was treated with 0.1 mL of 6 mol/L NaOH solution for 3 days in the dark, after which the reaction was terminated by adding 0.1 mL of 6 mol/L HCl, and the solution was then diluted to 0.5 mg/mL with acetonitrile. Prior to injection, all samples were subjected to filtration using 0.45 µm organic phase needle filters (ANPEL Laboratory Technologies Inc.), and were stored in amber vials. Following dissolution, samples were promptly analyzed.

### 3.4. LC-PDA Method Validation

Standard stock solution: A precise 5 mg of siponimod was measured and dissolved with 5 mL of acetonitrile to obtain a solution of 1 mg/mL.

Standard solution: 1 mL of the standard stock solution was accurately transferred into a 10 mL of volumetric flask and diluted with acetonitrile to a concentration of 100 µg/mL.

#### 3.4.1. Specificity

The specificity of the method was evaluated by injecting the blank solution and the test solutions into the column. Forced degradation samples were analyzed to assess separation between siponimod and its DPs. The PDA-based peak purity of siponimod and its DPs was evaluated to ensure no co-eluting species interfered with siponimod detection.

#### 3.4.2. LOD and LOQ

Standard siponimod solutions of varying concentrations were prepared through incremental dilution from the 100 µg/mL standard solution. The LOD and LOQ for siponimod were established at signal-to-noise ratios (S/N) of approximately 3:1 and 10:1, respectively.

#### 3.4.3. Linearity

Calibration solutions of six siponimod concentrations (1.062, 2.124, 5.310, 10.62, 106.2 and 265.5 µg/mL) were obtained by serial dilution of the standard stock solution. A calibration curve was then generated by plotting the peak area versus concentration, utilizing the least-squares approach. Residual analysis was examined to assess the accuracy of the linear regression model.

#### 3.4.4. Accuracy

Sample solution: An accurate amount of 1 mg of siponimod was weighed and introduced into 1 mL of the standard stock solution, followed by dilution with acetonitrile to a final volume of 10 mL, yielding a spiked concentration of 100 µg/mL. The mixture was sonicated for 15 min to ensure complete dissolution. Subsequently, the solution was filtered through a 0.45 µm organic phase filter membrane. Six independent samples at this concentration were prepared.

Accuracy was assessed by calculating the recovery of each sample relative to the standard solution, followed by the determination of the average recovery and RSD.

#### 3.4.5. Precision and Solution Stability

Intra-day precision was assessed by analyzing the peak areas obtained from six consecutive injections of the 100 µg/mL standard solution within the same laboratory. To evaluate inter-day precision, measurements were conducted on the standard solution over a span of three days. Additionally, the stability of the solution was evaluated by storing the standard solution at ambient temperature for 72 h.

#### 3.4.6. Robustness

The robustness of the method was evaluated by altering key parameters, including the flow rate (0.4 and 0.6 mL/min), the pH of the mobile phase (9.2 and 9.8), and the column temperature (25 °C and 35 °C). Variations in peak area, resolution, and retention time were systematically recorded and analyzed to determine the method’s stability under minor deviations from the optimal conditions.

#### 3.4.7. Acceptance Criteria for Each Validation Parameter

In accordance with the ICH Q2 (R2) guideline, method validation was performed for key analytical parameters. Table 7 summarizes analytical parameters and their corresponding acceptance criteria.

### 3.5. In Silico Toxicity Analysis

The toxicity of the DPs was assessed through two complementary web-based prediction tools, ProTox 3.0 [32] and ADMETlab 3.0 [33], with predictions for carcinogenicity and mutagenicity conducted.

### 3.6. Molecular Docking and MD Simulations

Molecular docking was conducted with AutoDock 4.2.6 [42] to explore the binding affinities and interaction patterns of siponimod and its DPs with the human sphingosine-1-phosphate receptor 1 (S1P1, PDB ID: 7EVY), a critical target in multiple sclerosis therapy [38]. The receptor model was constructed by eliminating crystallographic water molecules and non-essential heteroatoms, followed by the incorporation of polar hydrogens and the assignment of Gasteiger charges. Subsequently, energy minimization was performed to alleviate steric hindrance and achieve a stable conformation suitable for docking studies. Ligands, including siponimod and its DPs, were prepared by generating their three-dimensional structures, assigning partial charges, and specifying rotatable bonds. A grid box encompassing the receptor’s binding site was established to facilitate precise ligand positioning. Docking procedures were conducted utilizing the Lamarckian genetic algorithm, with binding affinities assessed based on computed docking energies and interaction analyses [43]. Three-dimensional visualization was accomplished using PyMOL 3.5.1, while Ligplus was employed to produce an “eyelash map”, allowing detailed mapping of hydrogen bonds, hydrophobic interactions, and other non-covalent contacts.

MD simulations were carried out using GROMACS 2023.5 [44]. Sobtop was employed to generate topol files for siponimod and its DPs. Protein–ligand complexes obtained from docking studies were immersed in a dodecahedral solvent box containing TIP3P water molecules and neutralized with appropriate counterions. Energy minimization was achieved via the steepest descent algorithm to eliminate steric conflicts. Subsequently, the systems were equilibrated under NVT and NPT conditions for 1 ns each at 300 K and 1 bar. A 100 ns production simulation was then performed, with trajectory snap-shots recorded every 10 ps. By analyzing the RMSD, RMSF, SASA, number of hydrogen bonds, and Rg of the protein backbone, combined with 3D free energy morphology diagrams, a comprehensive analysis was conducted to systematically dissect the structural stability of the complex, the motion characteristics of protein residues, the state of intermolecular interface interactions, and the spatial conformation features [45]. This provided reliable molecular dynamics data to elucidate the binding activity and interaction mechanism between small molecules and proteins.

## 4. Conclusions

A systematic forced degradation study of siponimod was carried out under various stress conditions, and it was found that siponimod in solid state remained stable under photolytic conditions, but was sensitive to acid, alkali, oxidation, and heat. A total of five DPs were isolated using a novel HPLC method, and their structures were elucidated through LC-HRMS/MS and LC-MS^n^ analysis.

DP-1 was an acidic degradation product. DP-2 and DP-3 were recognized as oxidative degradation products. DP-4 was a hydrolysis product generated under acidic, alkaline, or oxidative conditions. DP-5 was observed when siponimod was subjected to heating. Proposed degradation pathways for siponimod were outlined, and a thorough analysis revealed the primary degradation sites as follows: (1) hydrolysis of the imine functional group, (2) oxidation of the cyclobutane ring and the nitrogen atoms within the cyclobutane structure, (3) ring-opening of the azetidine ring. The in silico toxicity prediction suggested that siponimod and its DPs did not exhibit carcinogenic or mutagenic potential, implying that they were unlikely to contribute to additional safety risks associated with the drug. Additionally, the molecular docking analysis revealed that DP-2 and DP-3 exhibited moderately higher affinity than siponimod for binding to the S1P1 receptor, while DP-1 displayed a less favorable binding profile. MD simulations revealed that siponimod and its DPs consistently formed stable complexes with the S1P1 receptor over the 100 ns trajectory, maintaining both structural integrity and binding stability, which indicated favorable thermodynamic properties.

Overall, the results provide critical insights into the origin, potential toxicity, and biological activity of the DPs of siponimod, which may offer valuable clues for development of novel therapeutics with enhanced safety and efficacy.

## Figures and Tables

**Table 1:** The degradation conditions of siponimod in solid state and the DPs generated.

	Degradation Condition	DPs (% Area at 254 nm)
Oxidative	3% H_2_O_2_, 24 h	DP-2 (3.82%), DP-3 (30.73%), DP-4 (7.37%)
Acidic hydrolysis	6 mol/L HCl, 3 d	DP-1 (2.83%), DP-4 (7.81%)
Alkaline hydrolysis	6 mol/L NaOH, 3 d	DP-4 (6.99%)
Thermal	80 °C, 24 h	DP-5 (10.48%)
Photolytic	5000 lux, 10 d (a total irradiance of 1.2 × 10^6^ lux·hr)	/

**Table 2:** Accurate mass data of siponimod and its DPs in positive ion mode.

	Observed Mass (*m*/*z*)	Calculated Mass (*m*/*z*)	Proposed Molecular Formula	Error (ppm)	Proposed Loss
Siponimod	517.2665	517.2673	C_29_H_36_F_3_N_2_O_3_^+^	−1.55	
	416.2184	416.2196	C_25_H_29_F_3_NO^+^	−2.88	−C_4_H_7_NO_2_
	348.1559	348.1570	C_20_H_21_F_3_NO^+^	−3.16	−C_9_H_15_NO_2_
	241.1190	241.1199	C_14_H_16_F_3_^+^	−3.73	−C_15_H_20_N_2_O_3_
	178.1223	178.1226	C_11_H_16_NO^+^	−1.68	−C_18_H_20_F_3_NO_2_
	159.0412	159.0416	C_8_H_6_F_3_^+^	−2.52	−C_21_H_30_N_2_O_3_
DP-1	262.1429	262.1438	C_15_H_20_NO_3_^+^	−3.43	
	161.0953	161.0961	C_11_H_13_O^+^	−4.97	−C_4_H_7_NO_2_
	143.0851	143.0855	C_11_H_11_^+^	−2.80	−C_4_H_9_NO_3_
	128.0618	128.0621	C_10_H_8_^+•^	−2.34	−C_5_H_12_NO_3_•
	105.0699	105.0699	C_8_H_9_^+^	0	−C_7_H_11_NO_3_

**Table 3:** Accurate mass data of siponimod and its DPs in negative ion mode.

	Observed Mass (*m*/*z*)	Calculated Mass (*m*/*z*)	Proposed Molecular Formula	Error (ppm)	Proposed Loss
Siponimod	515.2518	515.2527	C_29_H_34_F_3_N_2_O_3_^−^	−1.75	
	243.1135	243.1139	C_14_H_15_N_2_O_2_^−^	−1.65	−C_15_H_19_F_3_O
	237.1094	237.1096	C_14_H_15_F_2_O^−^	−0.84	−C_15_H_19_FN_2_O_2_
	227.1054	227.1053	C_13_H_14_F_3_^−^	0.44	−C_16_H_20_N_2_O_3_
	217.1034	217.1034	C_14_H_14_FO^−^	0	−C_15_H_20_F_2_N_2_O_2_
	171.0926	171.0928	C_11_H_11_N_2_^−^	−1.17	−C_18_H_23_F_3_O_3_
	100.0404	100.0404	C_4_H_6_NO_2_^−^	0	−C_25_H_28_F_3_NO
	71.0138	71.0139	C_3_H_3_O_2_^−^	−1.41	−C_26_H_31_F_3_N_2_O
DP-1	260.1290	260.1292	C_15_H_18_NO_3_^−^	−0.77	
	188.1080	188.1081	C_12_H_14_NO^−^	−0.53	−C_3_H_4_O_2_
	161.0970	161.0972	C_11_H_13_O^−^	−1.24	−C_4_H_5_NO_2_
	100.0404	100.0404	C_4_H_6_NO_2_^−^	0	−C_11_H_12_O
	71.0140	71.0139	C_3_H_3_O_2_^−^	1.41	−C_12_H_15_NO
DP-2	531.2477	531.2476	C_29_H_34_F_3_N_2_O_4_^−^	0.19	
	237.1096	237.1096	C_14_H_15_F_2_O^−^	0	−C_15_H_19_FN_2_O_3_
	227.1051	227.1053	C_13_H_14_F_3_^−^	−0.88	−C_16_H_20_N_2_O_4_
	217.1030	217.1034	C_14_H_14_FO^−^	−1.84	−C_15_H_20_F_2_N_2_O_3_
	158.0974	158.0975	C_11_H_12_N^−^	−0.63	−C_18_H_22_F_3_NO_4_
	115.0275	115.0275	C_4_H_5_NO_3_^−•^	0	−C_25_H_29_F_3_NO•
	87.0088	87.0088	C_3_H_3_O_3_^−^	0	−C_26_H_31_F_3_N_2_O
	71.0139	71.0139	C_3_H_3_O_2_^−^	0	−C_26_H_31_F_3_N_2_O_2_
DP-3	531.2473	531.2476	C_29_H_34_F_3_N_2_O_4_^−^	−0.56	
	237.1095	237.1096	C_14_H_15_F_2_O^−^	−0.42	−C_15_H_19_FN_2_O_3_
	227.1053	227.1053	C_13_H_14_F_3_^−^	0	−C_16_H_20_N_2_O_4_
	217.1036	217.1034	C_14_H_14_FO^−^	0.92	−C_15_H_20_F_2_N_2_O_3_
	158.0971	158.0975	C_11_H_12_N^−^	−2.53	−C_18_H_22_F_3_NO_4_
	115.0275	115.0275	C_4_H_5_NO_3_^−•^	0	−C_25_H_29_F_3_NO•
	100.0405	100.0404	C_4_H_6_NO_2_^−^	1.00	−C_25_H_28_F_3_NO_2_
	87.0087	87.0088	C_3_H_3_O_3_^−^	−1.15	−C_26_H_31_F_3_N_2_O
	71.0138	71.0139	C_3_H_3_O_2_^−^	−1.41	−C_26_H_31_F_3_N_2_O_2_
DP-4	533.2634	533.2633	C_29_H_36_F_3_N_2_O_4_^−^	0.19	
	503.2531	503.2527	C_28_H_34_F_3_N_2_O_3_^−^	0.79	−CH_2_O
	261.1247	261.1245	C_14_H_17_N_2_O_3_^−^	0.77	−C_15_H_19_F_3_O
	237.1096	237.1096	C_14_H_15_F_2_O^−^	0	−C_15_H_21_FN_2_O_3_
	231.1136	231.1139	C_13_H_15_N_2_O_2_^−^	−1.30	−C_16_H_21_F_3_O_2_
	227.1049	227.1053	C_13_H_14_F_3_^−^	−1.76	−C_16_H_22_N_2_O_4_
	217.1034	217.1034	C_14_H_14_FO^−^	0	−C_15_H_22_F_2_N_2_O_3_
	118.0509	118.0510	C_4_H_8_NO_3_^−^	−0.85	−C_25_H_28_F_3_NO
	88.0404	88.0404	C_3_H_6_NO_2_^−^	0	−C_26_H_30_F_3_NO_2_
	71.0139	71.0139	C_3_H_3_O_2_^−^	0	−C_26_H_33_F_3_N_2_O_2_
DP-5	515.2521	515.2527	C_29_H_34_F_3_N_2_O_3_^−^	−1.16	
	243.1133	243.1139	C_14_H_15_N_2_O_2_^−^	−2.47	−C_15_H_19_F_3_O
	237.1095	237.1096	C_14_H_15_F_2_O^−^	−0.42	−C_15_H_19_FN_2_O_2_
	227.1051	227.1053	C_13_H_14_F_3_^−^	−0.88	−C_16_H_20_N_2_O_3_
	217.1033	217.1034	C_14_H_14_FO^−^	−0.46	−C_15_H_20_F_2_N_2_O_2_
	100.0404	100.0404	C_4_H_6_NO_2_^−^	0	−C_25_H_28_F_3_NO
	85.0296	85.0295	C_4_H_5_O_2_^−^	1.18	−C_25_H_29_F_3_N_2_O

**Table 4:** The in silico predictions of carcinogenic and mutagenic properties for siponimod and its DPs.

Peak Name	Carcinogenicity/Probability	Mutagenicity/Probability
Siponimod	Negative/0.55	Negative/0.60
DP-1	Negative/0.56	Negative/0.72
DP-2	Negative/0.58	Negative/0.60
DP-3	Negative/0.55	Negative/0.55
DP-4	Negative/0.56	Negative/0.56
DP-5	Negative/0.57	Negative/0.59

**Table 5:** The affinity of the ligands interacting with the S1P1 receptor.

Peak Name	Affinity/(kcal/mol)
Siponimod	−10.32
DP-1	−6.93
DP-2	−11.16
DP-3	−12.70
DP-4	−9.98
DP-5	−10.6

**Table 6:** The key S1P1 receptor amino acids interacting with DPs versus siponimod.

Interaction Type	Siponimod	DP-1	DP-2	DP-3	DP-4	DP-5
Hydrophobic Interactions	Phe125, Leu128, Val132, Leu195, Val209, Phe210, Leu272, Leu297	Leu128, Val209, Phe210, Trp269, Leu272, Phe273, Leu276	Met124, Phe125, Leu128, Val132, Phe133, Leu195, Val209, Phe210, Leu213, Leu276, Ala293, Glu294, Leu297	Phe125, Leu128, Val132, Leu195, Val209, Phe210, Leu272, Leu276, Leu297	Phe125, Leu128, Val132, Val194, Leu195, Val209, Phe210, Leu213, Trp269, Leu272, Ala293, Leu297	Phe125, Leu128, Val132, Leu195, Val209, Phe210, Leu272, Leu276, Glu294, Leu297
Hydrogen Bonds	Tyr29, Ser105, Thr109, Trp117	Ser129	Ala293, Glu294	Tyr29, Ser105, Thr109	Lys34, Val194, Ala293, Glu294	Val194, Phe291, Ala293, Glu294
Salt Bridges	Lys34, Arg120, Glu121	/	Lys34	Arg120, Glu121	Lys34	Lys34
Halogen Bonds	Cys206	/	Phe125	Cys206	Cys206	Cys206
π-Stacking	/	/	/	Trp269	/	Trp269

**Table 7:** Validation parameters and corresponding acceptance criteria.

Validation Parameter	Acceptance Criteria	Justification
Specificity	PDA peak purity value ≥ 0.95; resolution between siponimod and any degradation product ≥ 1.5	The method can accurately identify and quantify the analyte in the presence of potential impurities, degradation products, or other matrix components.
Accuracy	95–102% recovery for siponimod	Typical acceptance limits for drug substance assays
Intra-day precision	RSD ≤ 2.0%	The degree of similarity between peak area results of analytes over a short time interval
Inter-day precision	RSD ≤ 2.0%	The similarity level of peak area results for analytes from different dates within the same laboratory
Linearity	Regression coefficient (r) should be ≥0.999; residual plot shows random distribution.	Proportional relationship between concentration and response across the working range
Robustness	Resolution between siponimod and the adjacent impurity ≥ 1.5 under different conditions	Minor method deviations (e.g., temperature, solvent composition) do not significantly affect analytical outcomes.

## Data Availability

The original contributions presented in this study are included in the article/Supplementary Materials. Further inquiries can be directed to the corresponding authors.

## Supplementary Materials

The following supporting information can be downloaded at: https://www.mdpi.com/article/10.3390/ijms27083630/s1.

## Abbreviations

The following abbreviations are used in this manuscript:

S1P	Sphingosine 1-phosphate
SPMS	Secondary progressive multiple sclerosis
CNS	Central nervous system
DMT	Disease-modifying therapy
RRMS	Relapsing–remitting multiple sclerosis
API	Active pharmaceutical ingredient
RP-UPLC	Reversed-phase ultra-high-performance liquid chromatography
DP	Degradation product
LC-PDA	Liquid chromatography–photodiode array detector
LC-HRMS/MS	Liquid chromatography–high-resolution tandem mass spectrometry
LC-MS^n^	Liquid chromatography–multi-stage mass spectrometry
HPLC	High-performance liquid chromatography
ESI	Electrospray ionization
IS	Ionspray voltage
TEM	Source temperature
CUR	Curtain gas
EP	Entrance potential
DP	Declustering potential
CE	Collision energy
CXP	Collision cell exit potential
LOQs	Limits of quantitation
LODs	Limits of detection
S/Ns	Signal-to-noise ratios
PDB	Protein data bank
ICH	International council for harmonisation of technical requirements for pharmaceuticals for human use
MD	Molecular dynamics
RSD	Relative standard deviation
RMSD	Root-mean-square deviation
RMSF	Root-mean-square fluctuation
Rg	Radius of gyration
SAR	Structure–activity relationship
SASA	Solvent-accessible surface area
