# Supplementary material for: Structural Characterization, Toxicity Assessment and Molecular Modeling of Forced Degradation Products of Siponimod
Source: Int J Mol Sci. 2026 Apr 18;27(8):3630. doi: 10.3390/ijms27083630 (PMC13116541; doi:10.3390/ijms27083630)
Supplement: Supplementary file 1 [file ijms-27-03630-s001.zip › ijms-4183739-Supplementary Material.pdf]

Supplementary Material

Structural characterization, toxicity assessment and molecular modeling of forced degradation products of siponimod

Yajing Liang [1], Tingting Zhang [1], Dongfeng Zhang [1], Bo Jin [1,*] and Chen Ma [1,*]

[1] Institute of Materia Medica, Chinese Academy of Medical Sciences & Peking Union Medical College, Beijing 100050, China

* Correspondence: jinboyws@imm.ac.cn; mach@imm.ac.cn

Table of contents

Figure S1. PDA-based peak purity plots.

Figure S2. The residuals vs. concentration plot.

Figure S3. Primary mass spectrum of siponimod in ESI (+).

Figure S4. HRMS/MS spectrum of siponimod in ESI (+).

Figure S5. MS^3 spectrum of siponimod in ESI (+).

Figure S6. Primary mass spectrum of siponimod in ESI (-).

Figure S7. HRMS/MS spectrum of siponimod in ESI (-).

Figure S8. MS^3 spectrum of siponimod in ESI (-).

Figure S9. Primary mass spectrum of DP-1 in ESI (+).

Figure S10. HRMS/MS spectrum of DP-1 in ESI (+).

Figure S11. MS^3 spectra of DP-1 in ESI (+).

Figure S12. Primary mass spectrum of DP-1 in ESI (-).

Figure S13. HRMS/MS spectrum of DP-1 in ESI (-).

Figure S14. Primary mass spectrum of DP-2 in ESI (-).

Figure S15. HRMS/MS spectrum of DP-2 in ESI (-).

Figure S16. Primary mass spectrum of DP-3 in ESI (-).

Figure S17. HRMS/MS spectrum of DP-3 in ESI (-).

Figure S18. Primary mass spectrum of DP-4 in ESI (-).

Figure S19. HRMS/MS spectrum of DP-4 in ESI (-).

Figure S20. MS^3 spectrum of DP-4 in ESI (-).

Figure S21. Primary mass spectrum of DP-5 in ESI (-).

Figure S22. HRMS/MS spectrum of DP-5 in ESI (-).

Figure S23. Docking interaction of siponimod 3D and 2D diagrams.

Figure S24. Docking interaction of DP-1 3D and 2D diagrams.

Figure S25. Docking interaction of DP-2 3D and 2D diagrams.

Figure S26. Docking interaction of DP-4 3D and 2D diagrams.

Figure S27. Docking interaction of DP-5 3D and 2D diagrams.

Figure S28. Examination of MD simulations data of siponimod.

Figure S29. Examination of MD simulations data of DP-1.

Figure S30. Examination of MD simulations data of DP-2.

Figure S31. Examination of MD simulations data of DP-4.

Figure S32. Examination of MD simulations data of DP-5.

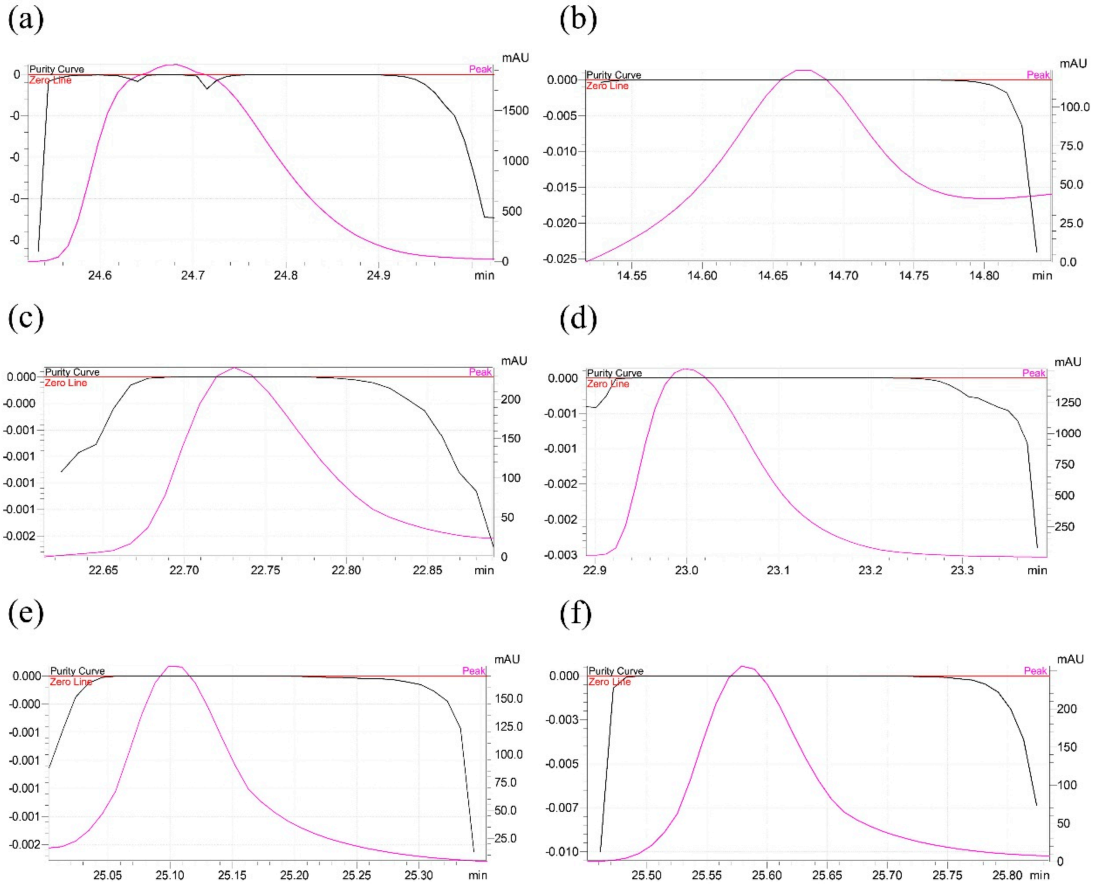

Figure S1. PDA-based peak purity plots: (**a**) Siponimod; (**b**) DP-1; (**c**) DP-2; (**d**) DP-3; (**e**) DP-4; (**f**) DP-5.

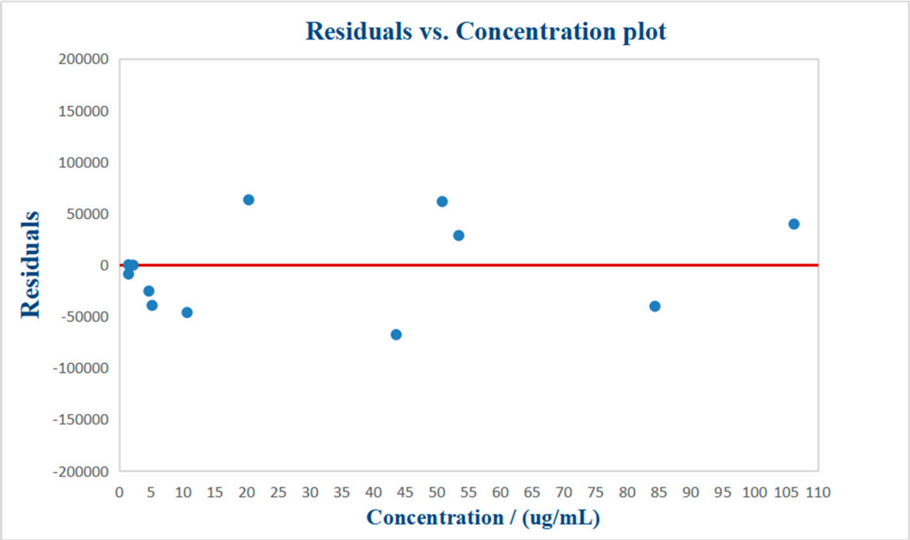

Figure S2. The residuals vs. concentration plot.

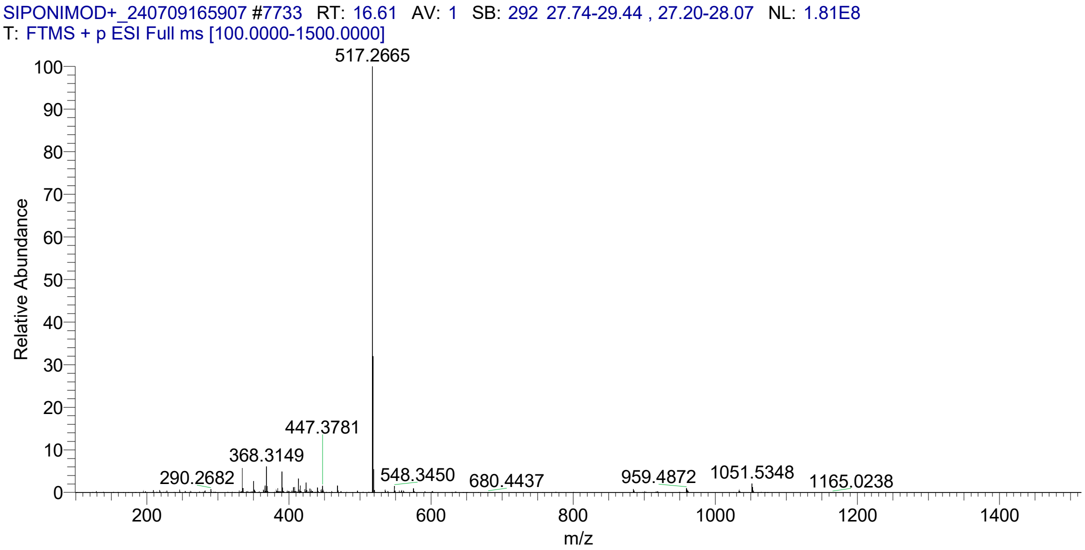

Figure S3. Primary mass spectrum of siponimod in ESI (+).

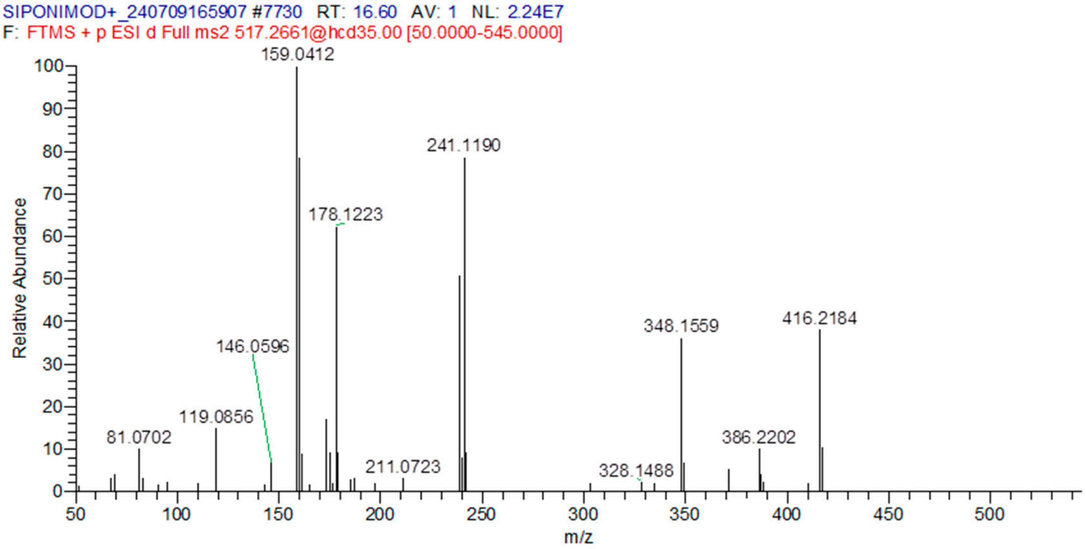

Figure S4. HRMS/MS spectrum of siponimod in ESI (+).

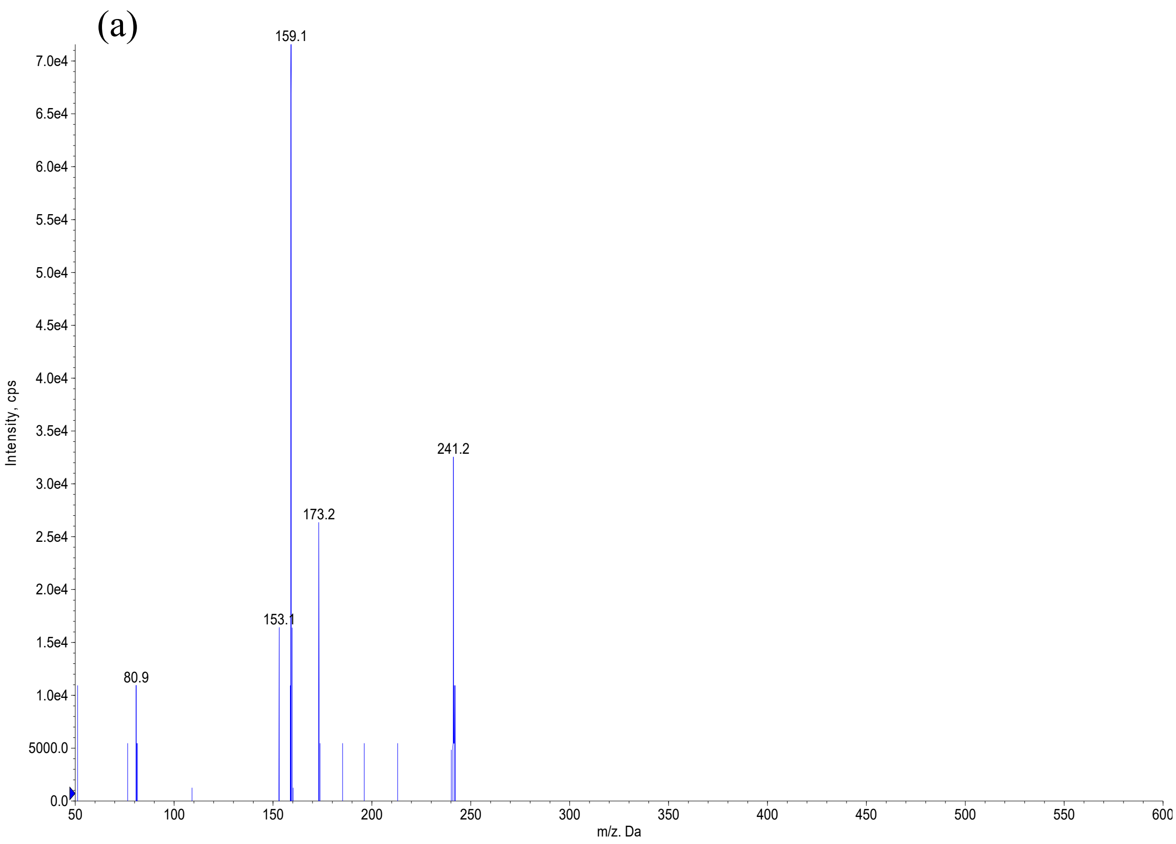

Figure S5. MS^3 spectrum of siponimod in ESI (+): (**a**) *m/z* 159 ion from *m/z* 241.

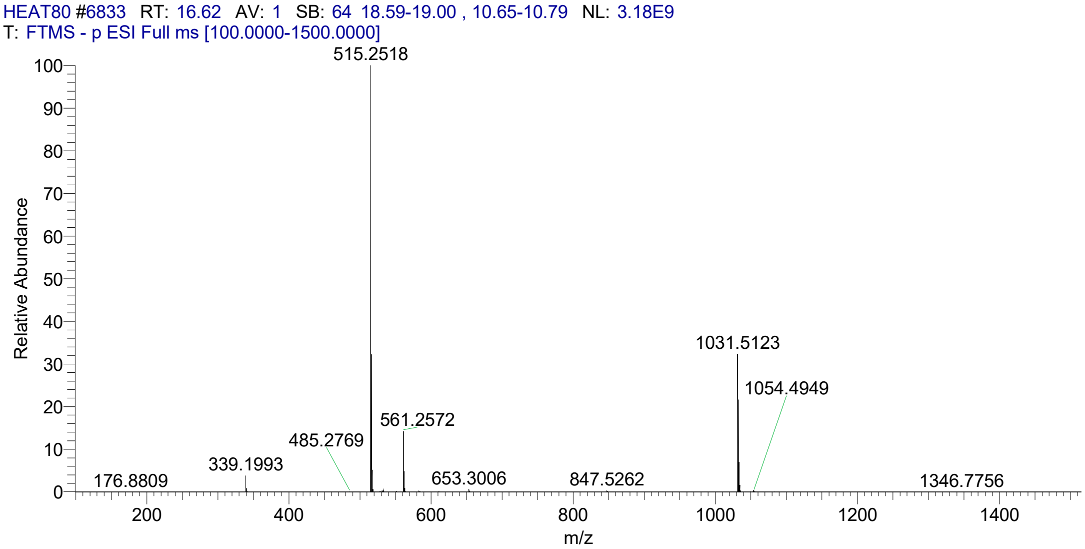

Figure S6. Primary mass spectrum of siponimod in ESI (-).

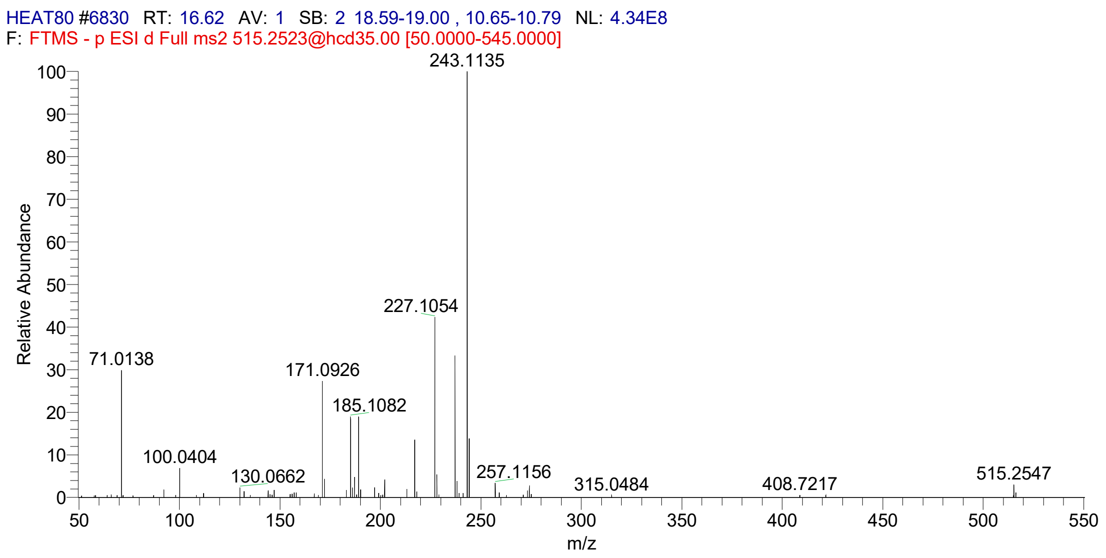

Figure S7. HRMS/MS spectrum of siponimod in ESI (-).

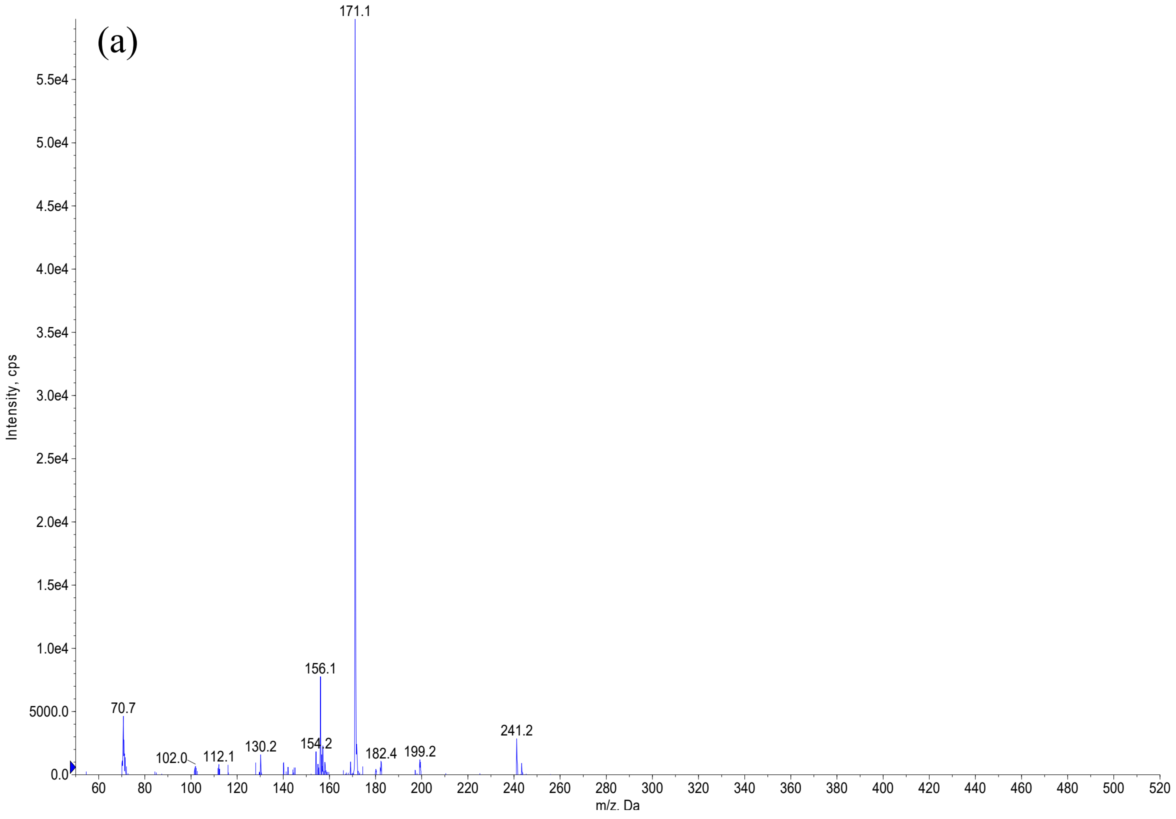

Figure S8. MS^3 spectrum of siponimod in ESI (-): (**a**) *m/z* 171 ion from *m/z* 243.

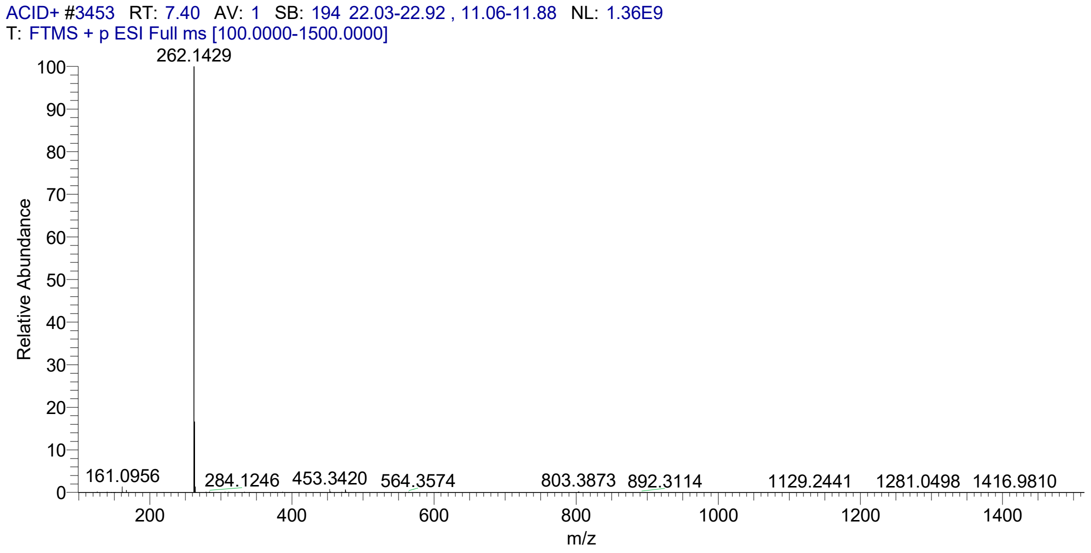

Figure S9. Primary mass spectrum of DP-1 in ESI (+).

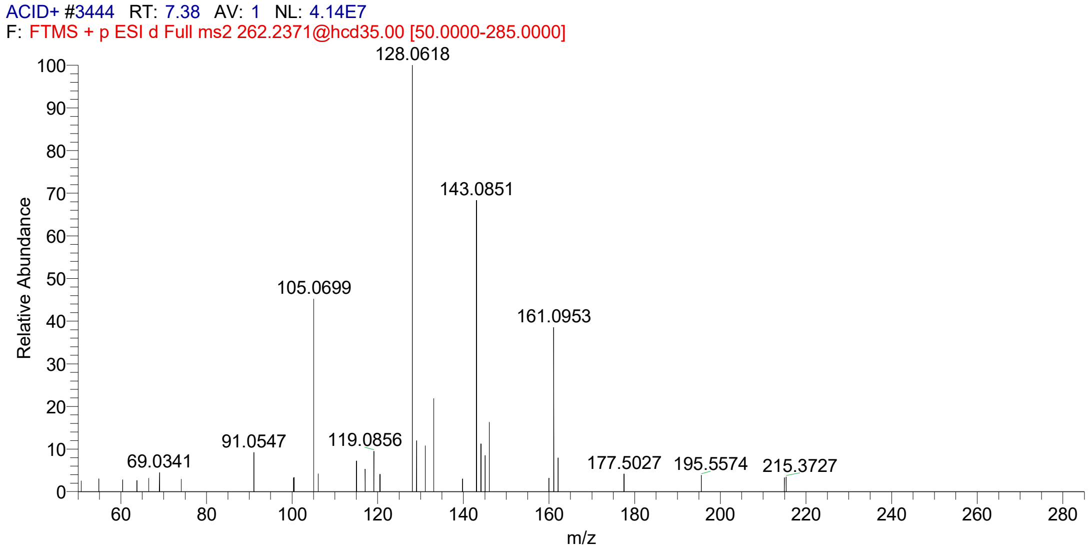

Figure S10. HRMS/MS spectrum of DP-1 in ESI (+).

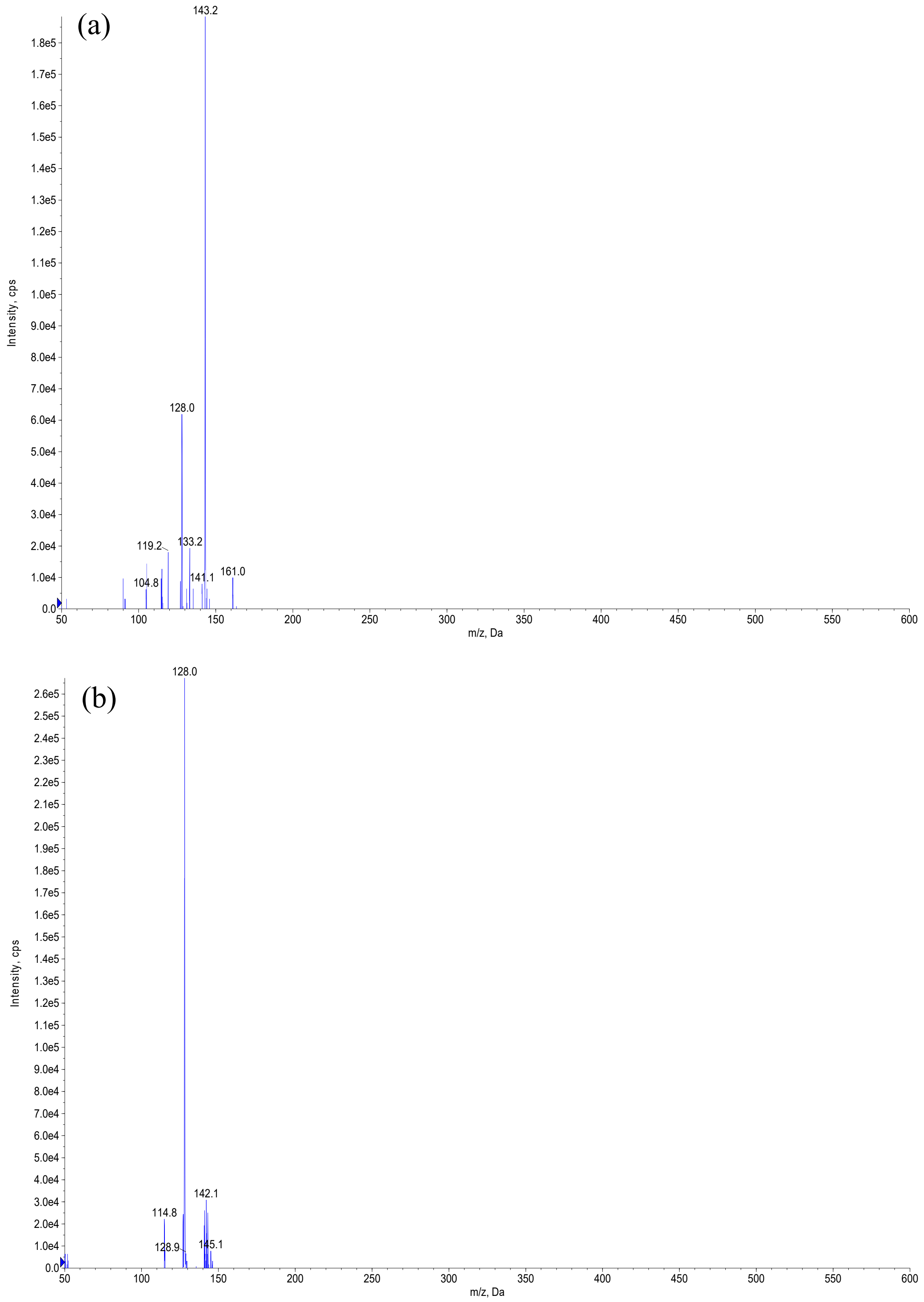

Figure S11. MS^3 spectra of DP-1 in ESI (+): (**a**) *m/z* 143 ion from *m/z* 161; (**b**) *m/z* 128 ion from *m/z* 143.

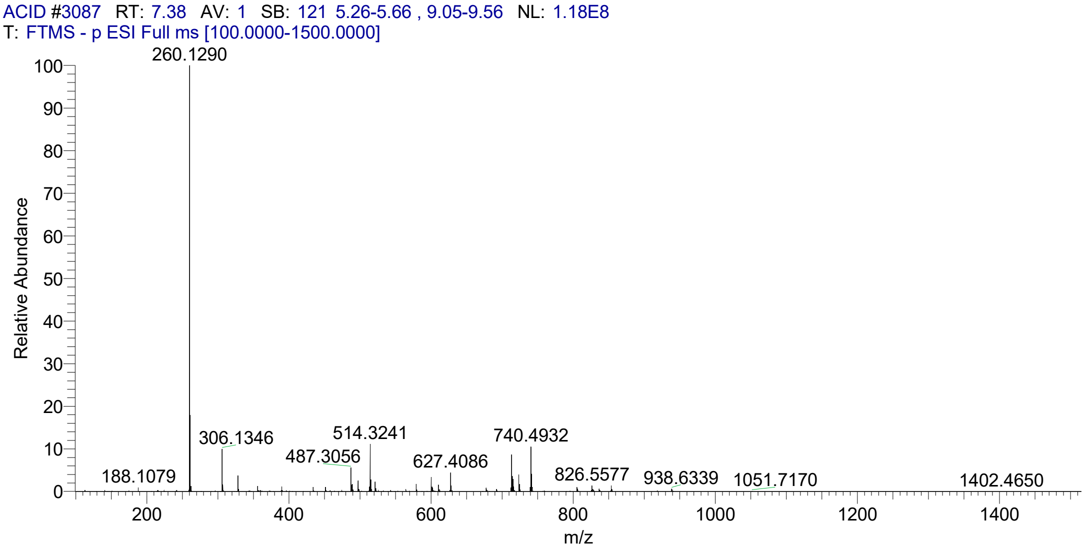

Figure S12. Primary mass spectrum of DP-1 in ESI (-).

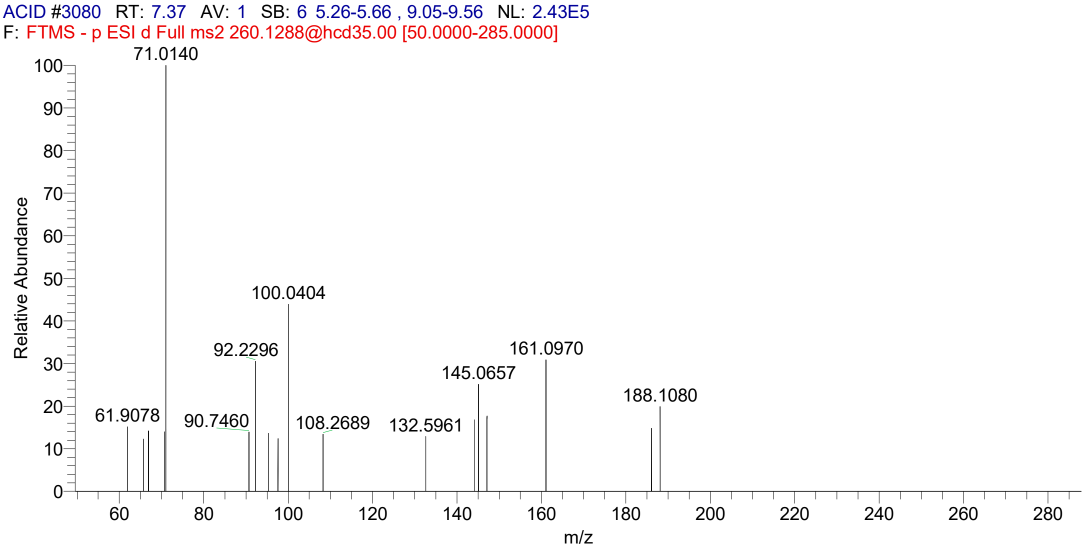

Figure S13. HRMS/MS spectrum of DP-1 in ESI (-).

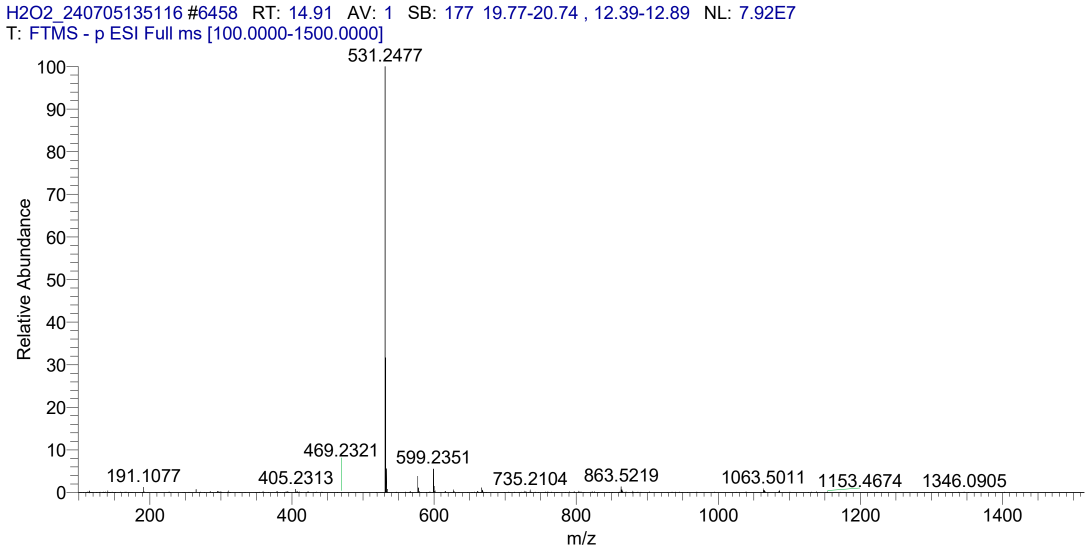

Figure S14. Primary mass spectrum of DP-2 in ESI (-).

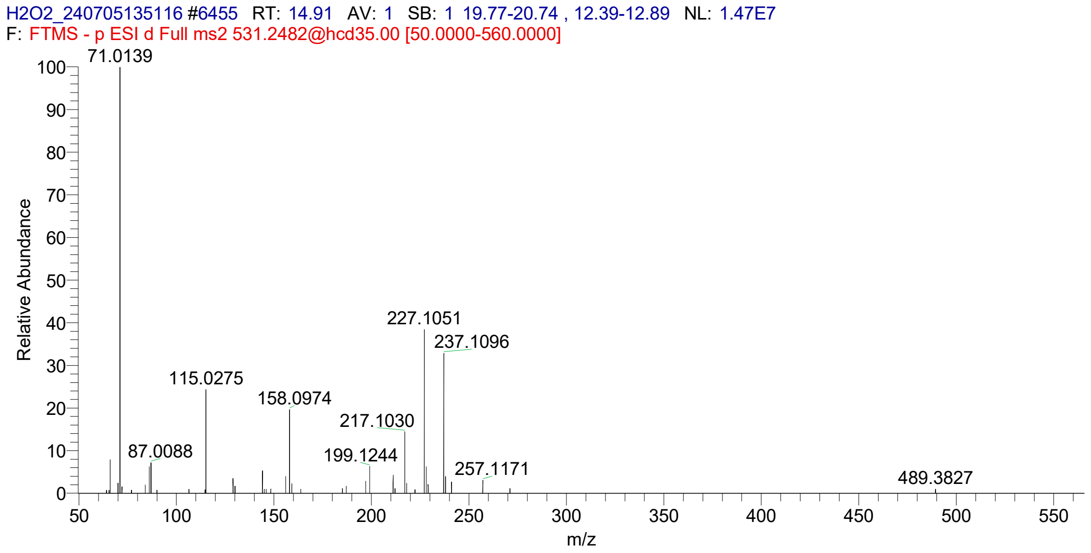

Figure S15. HRMS/MS spectrum of DP-2 in ESI (-).

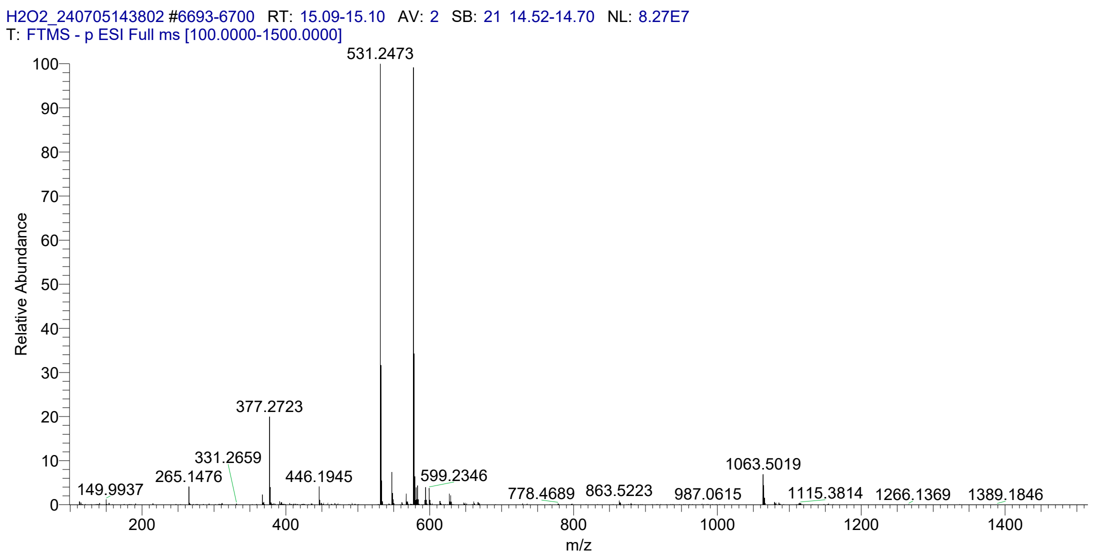

Figure S16. Primary mass spectrum of DP-3 in ESI (-).

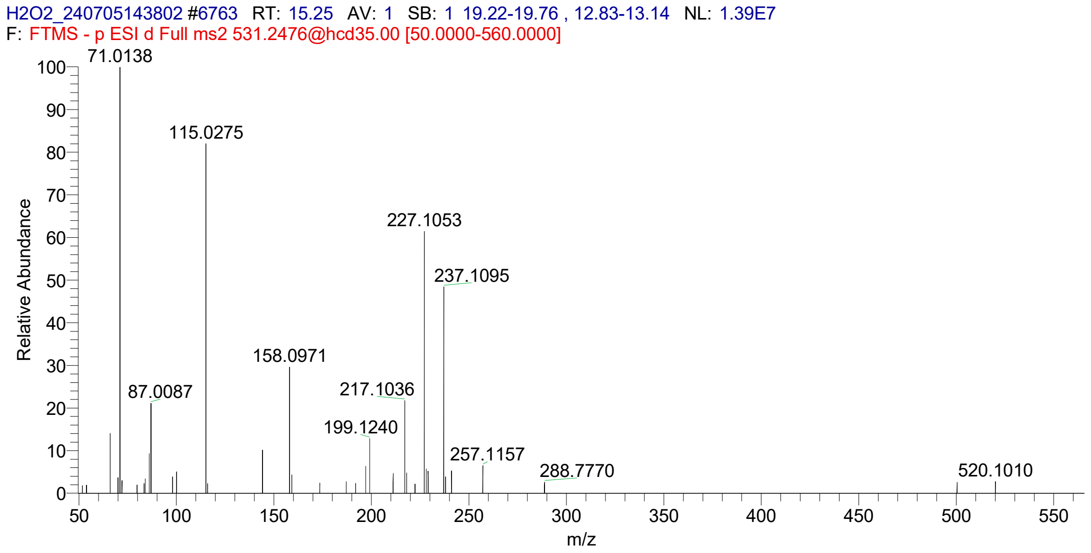

Figure S17. HRMS/MS spectrum of DP-3 in ESI (-).

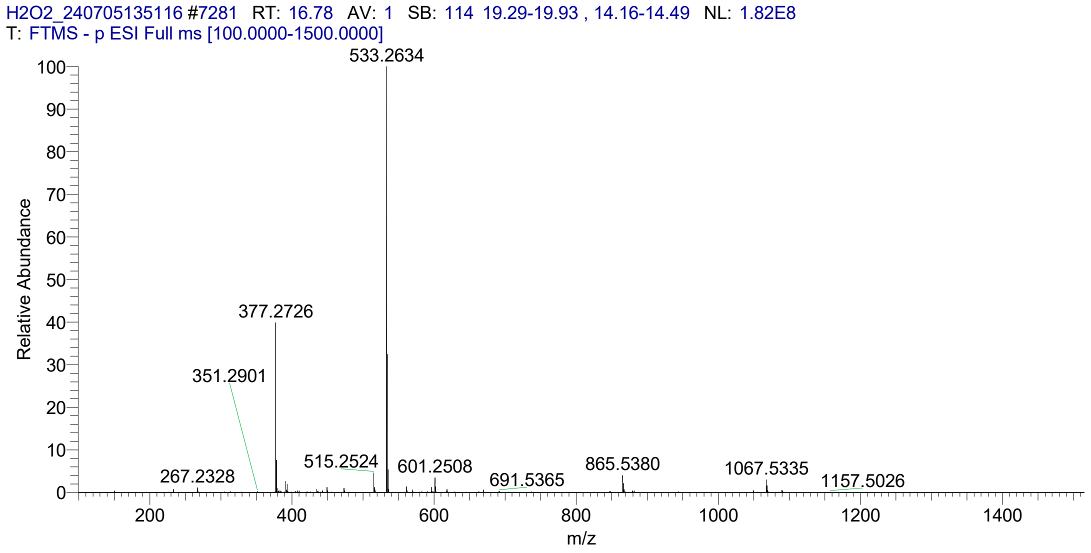

Figure S18. Primary mass spectrum of DP-4 in ESI (-).

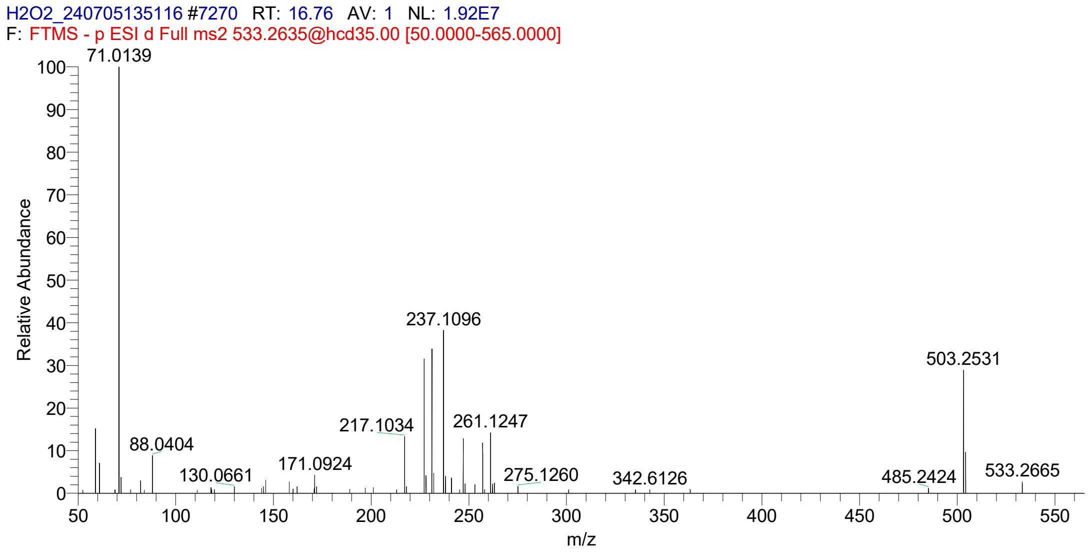

Figure S19. HRMS/MS spectrum of DP-4 in ESI (-).

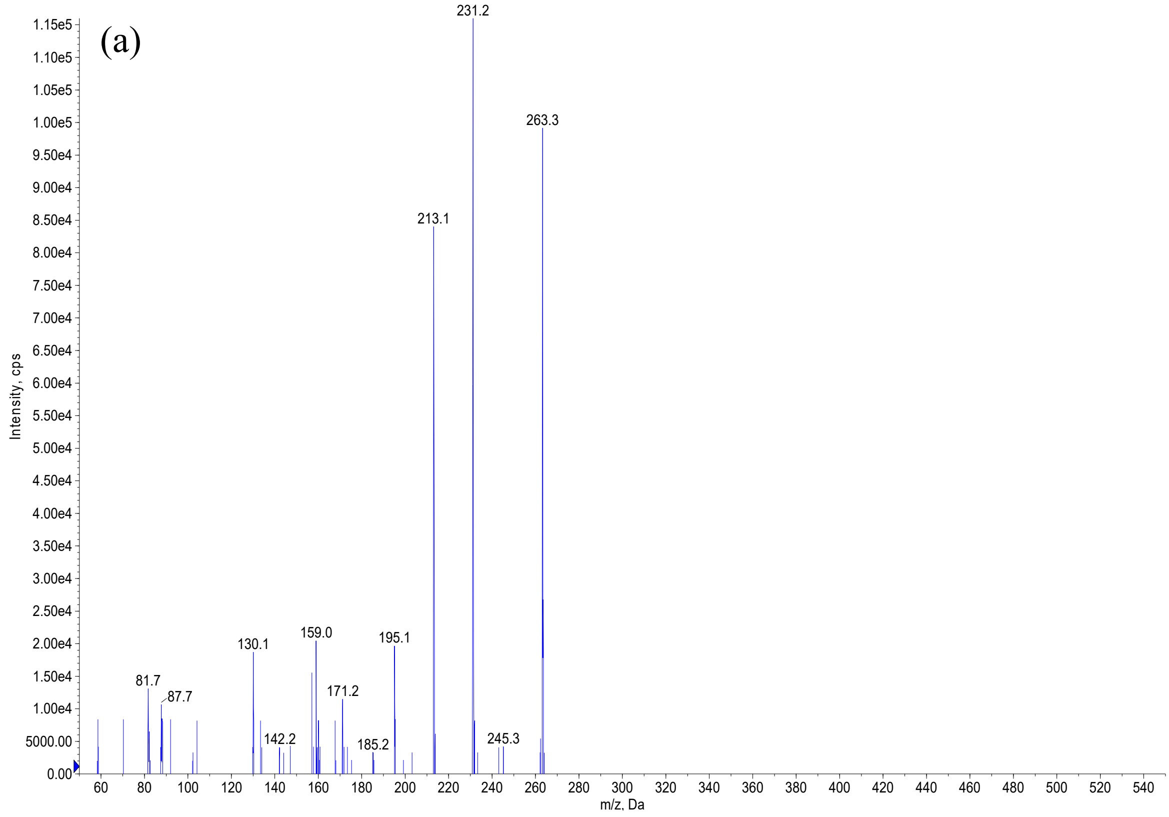

Figure S20. MS^3 spectrum of DP-4 in ESI (-): (**a**) *m*/*z* 231 ion from *m*/*z* 261.

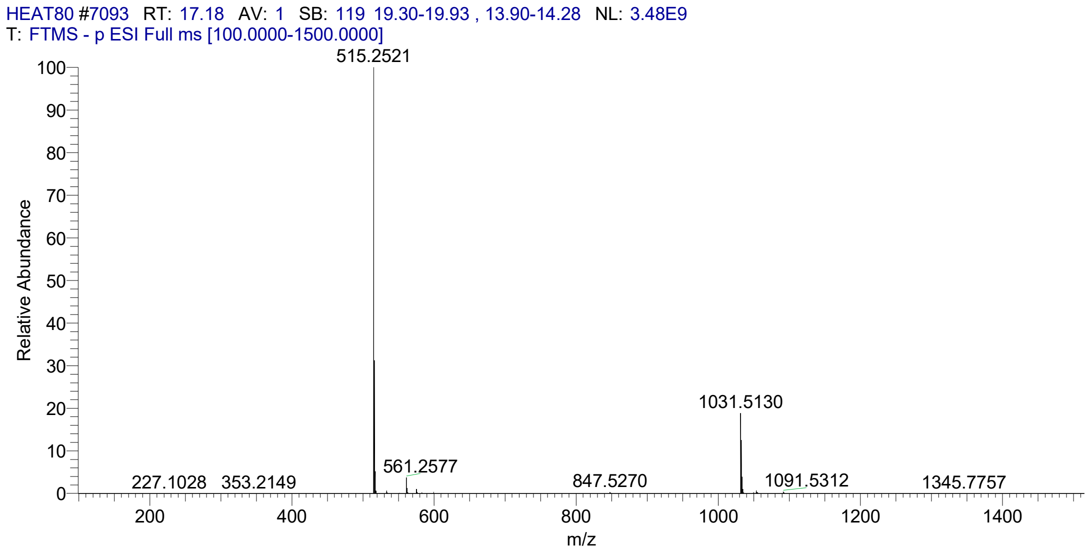

Figure S21. Primary mass spectrum of DP-5 in ESI (-).

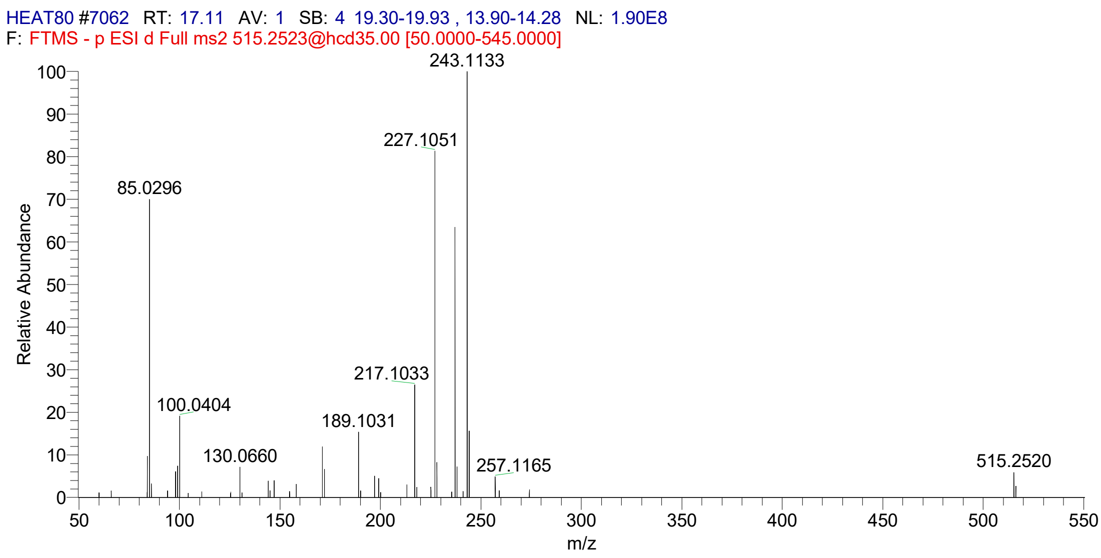

Figure S22. HRMS/MS spectrum of DP-5 in ESI (-).

(a)

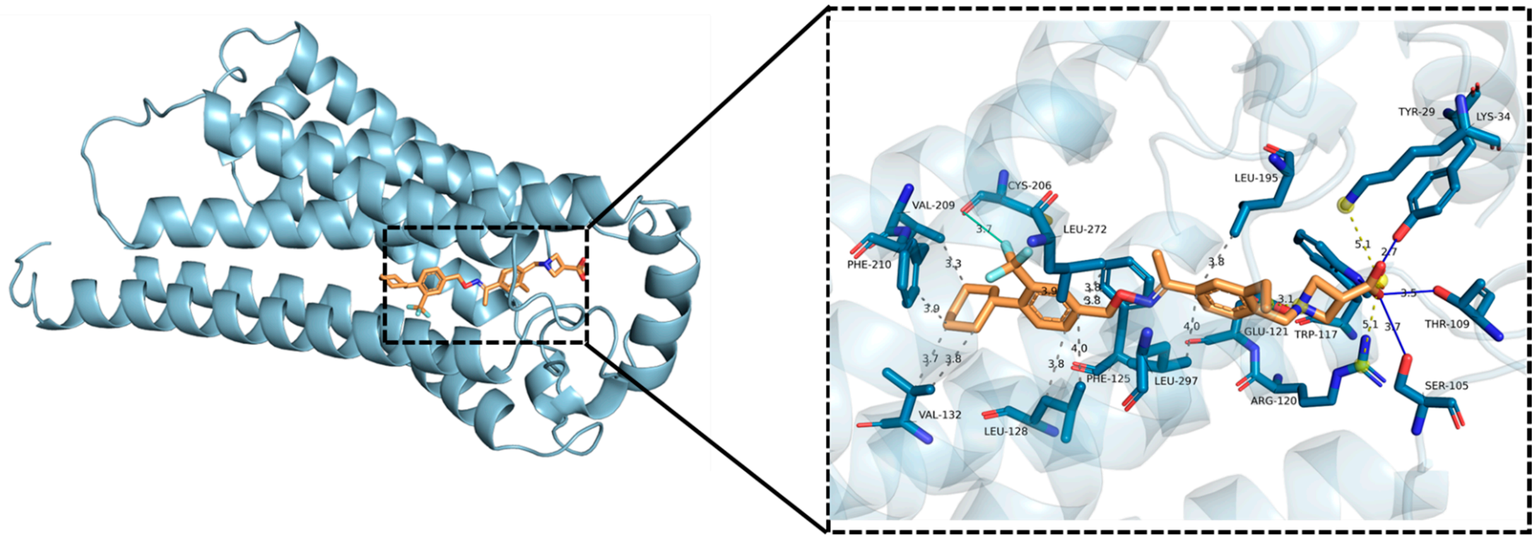

(b)

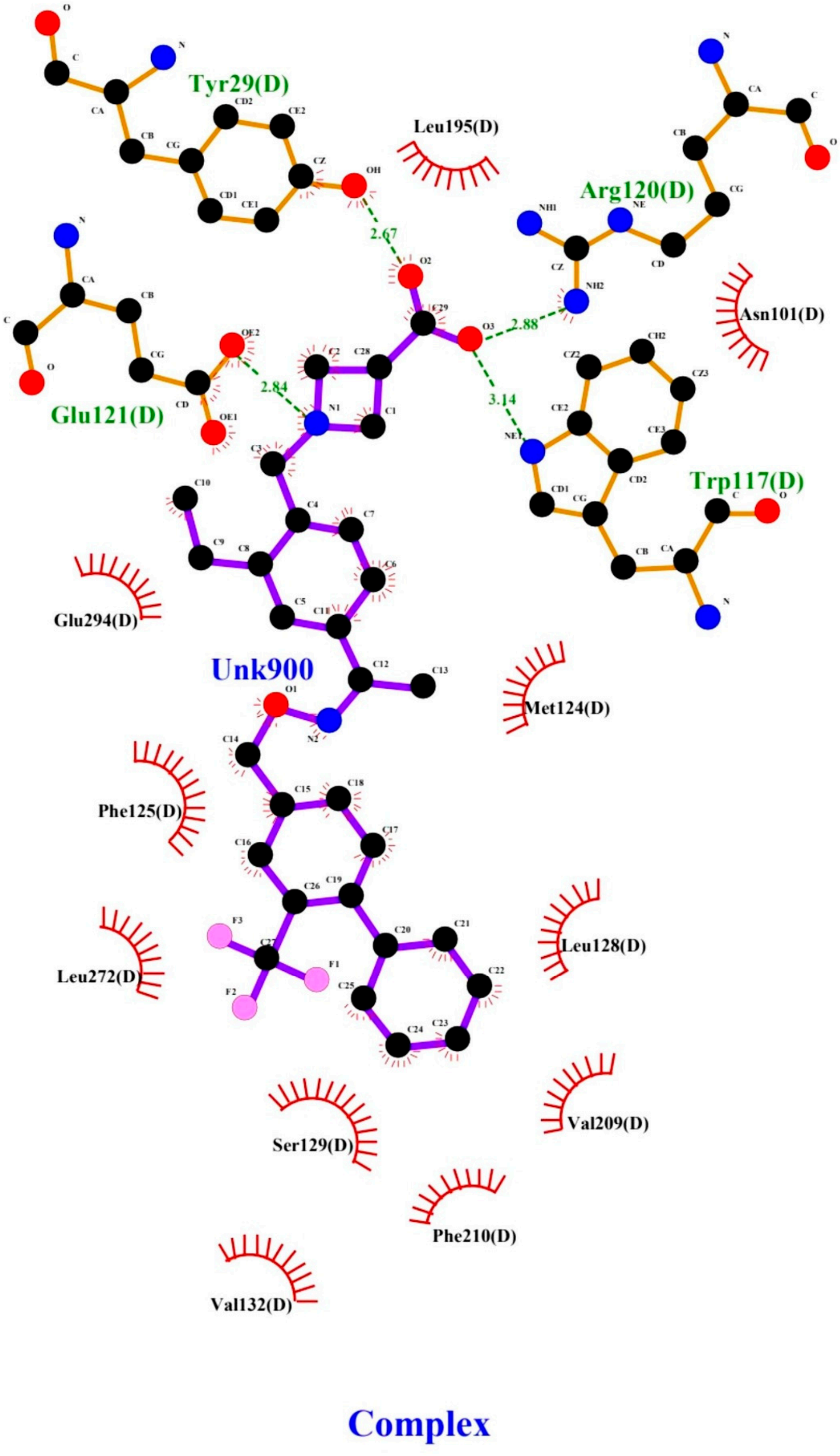

Figure S23. Docking interaction of siponimod 3D (**a**) and 2D (**b**) diagrams.

(a)

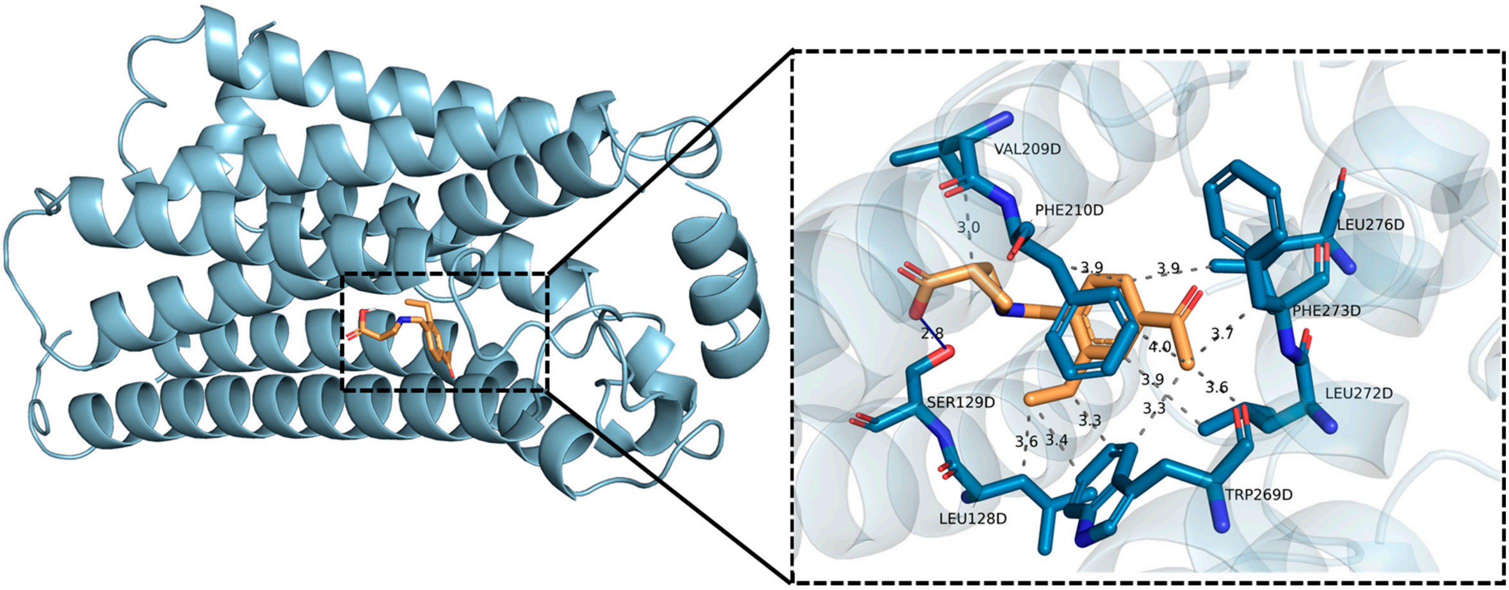

(b)

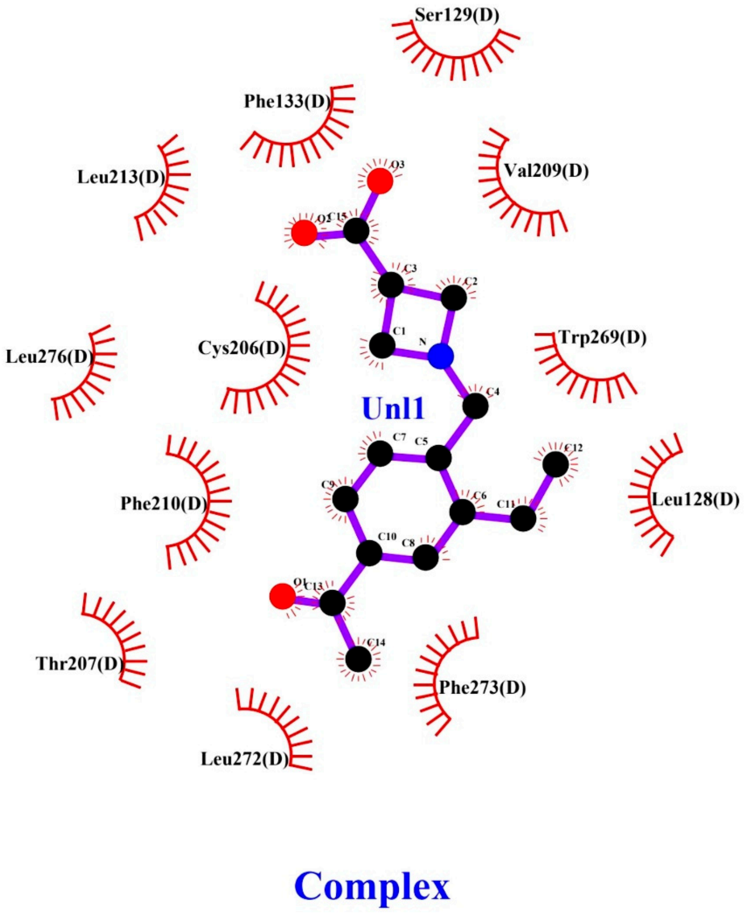

Figure S24. Docking interaction of DP-1 3D (**a**) and 2D (**b**) diagrams.

(a)

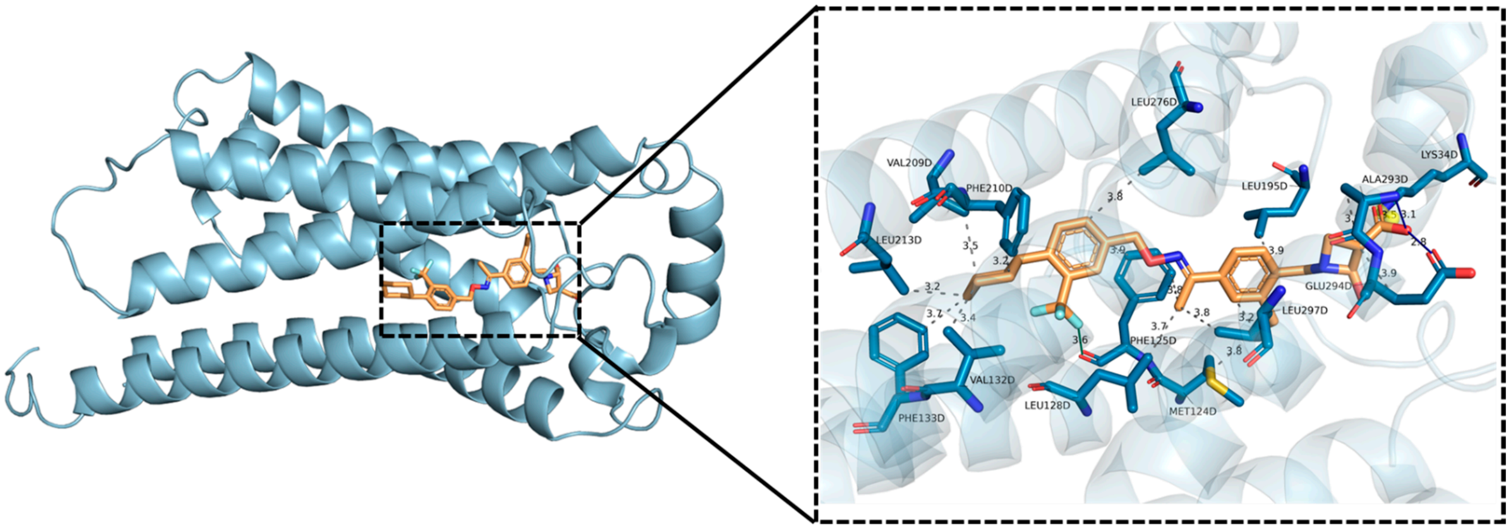
LEU276D
VAL209D
PHE210D
LYS34D
LEU195D
ALA293D
LEU213D
GLU294D
LEU297D
PHE125D
VAL132D
LEU128D
MET124D
PHE133D

(b)

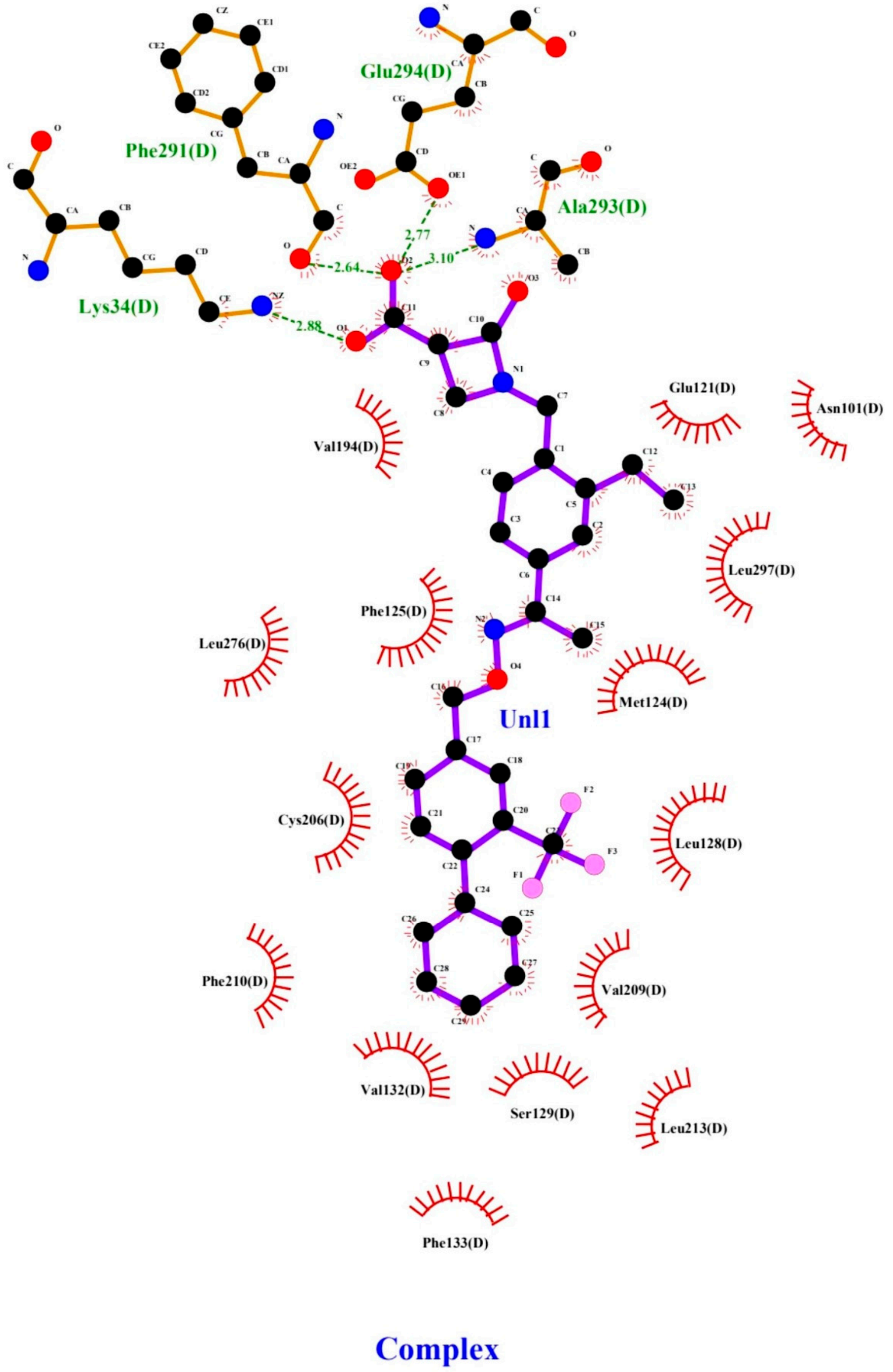

Figure S25. Docking interaction of DP-2 3D (**a**) and 2D (**b**) diagrams.

(a)

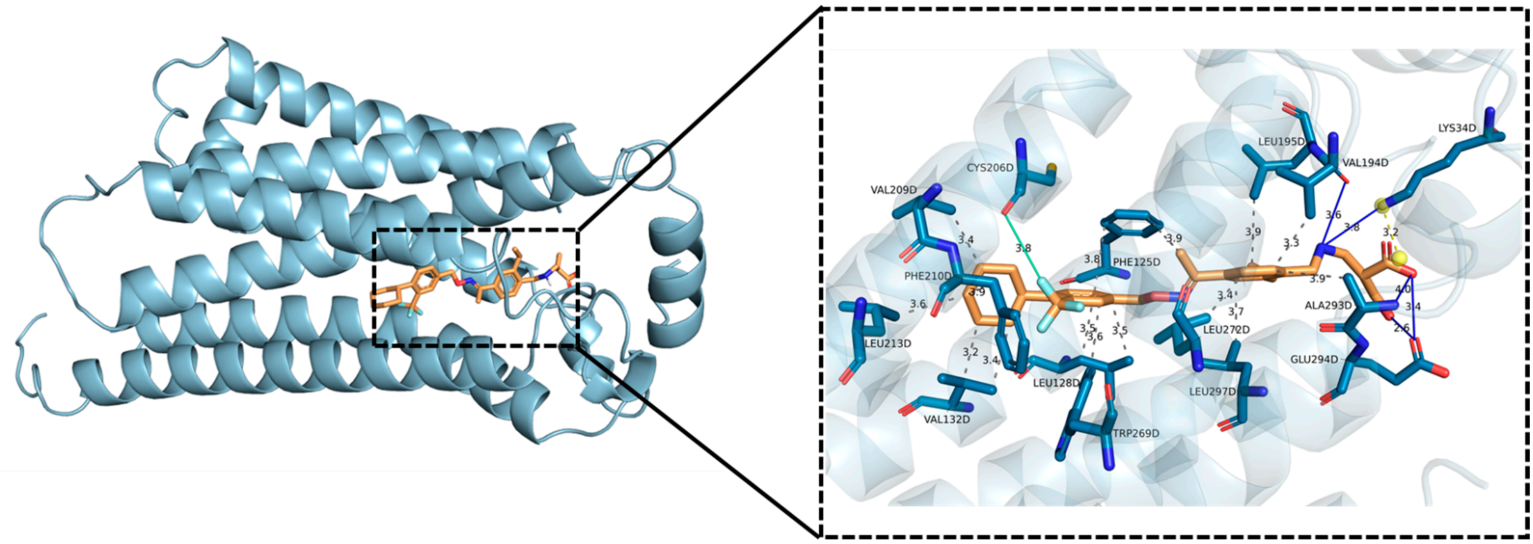

LEU195D
VAL194D
LYS34D
CYS206D
VAL209D
PHE210D
PHE125D
LEU213D
ALA293D
LEU272D
GLU294D
LEU128D
LEU297D
VAL132D
TRP269D

(b)

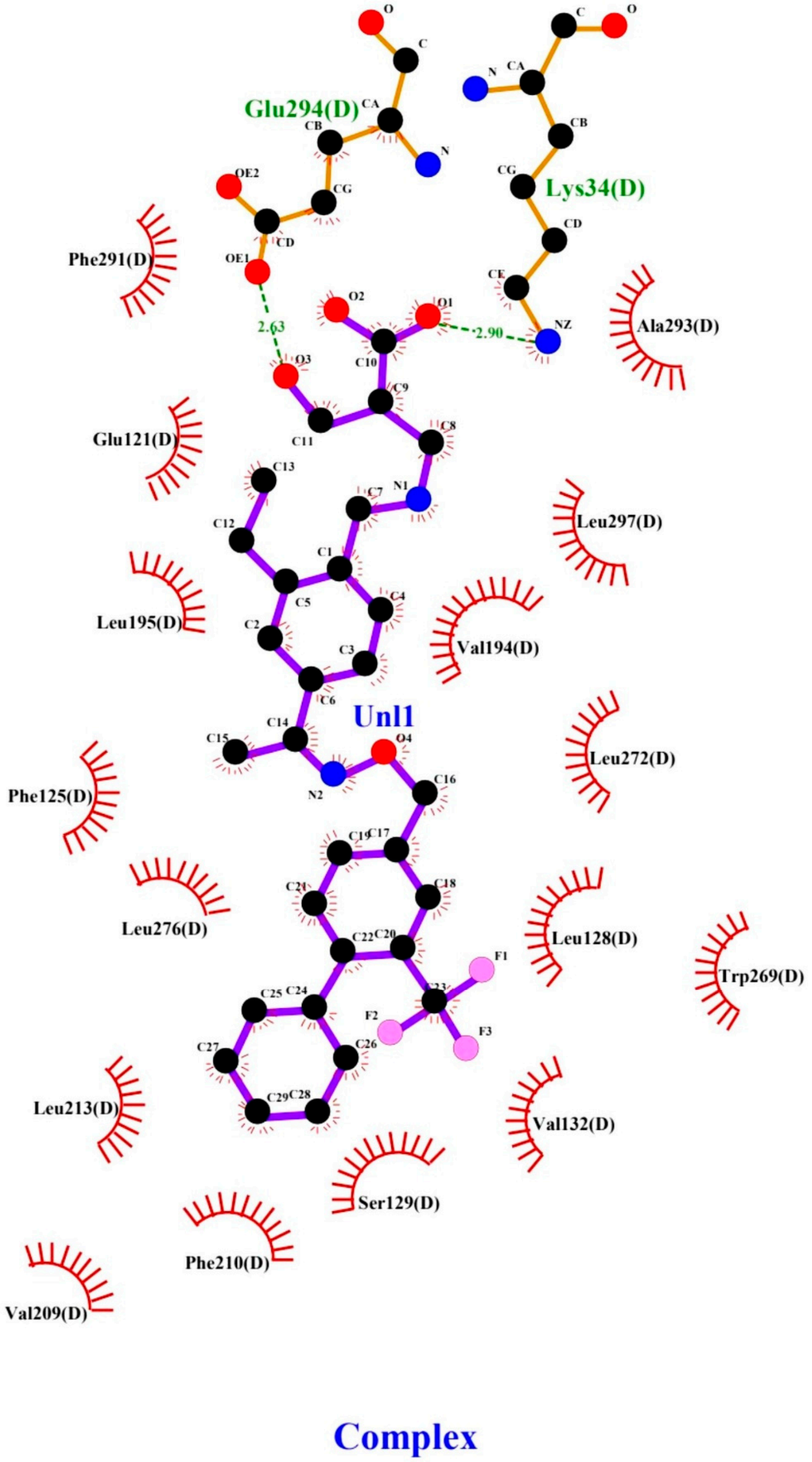

Figure S26. Docking interaction of DP-4 3D (**a**) and 2D (**b**) diagrams.

(a)

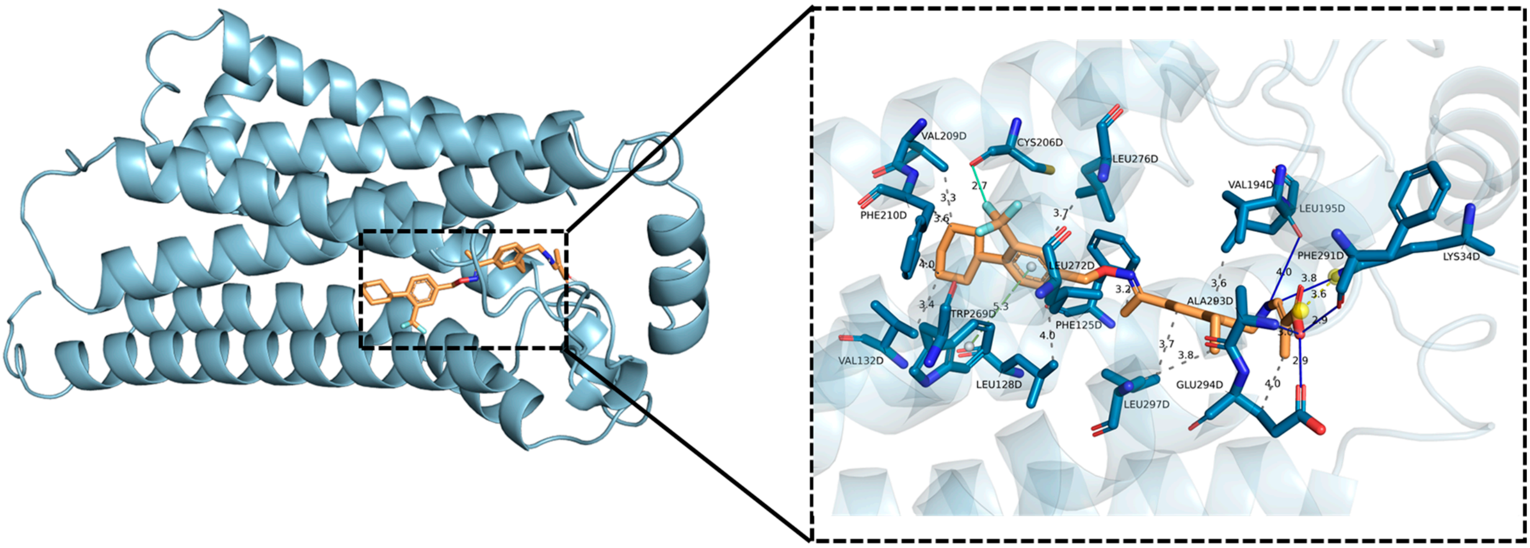
VAL209D
CYS206D
LEU276D
PHE210D
VAL194D
LEU195D
LEU272D
PHE291D
LYS34D
ALA293D
TRP269D
PHE125D
VAL132D
LEU128D
GLU294D
LEU297D

(b)

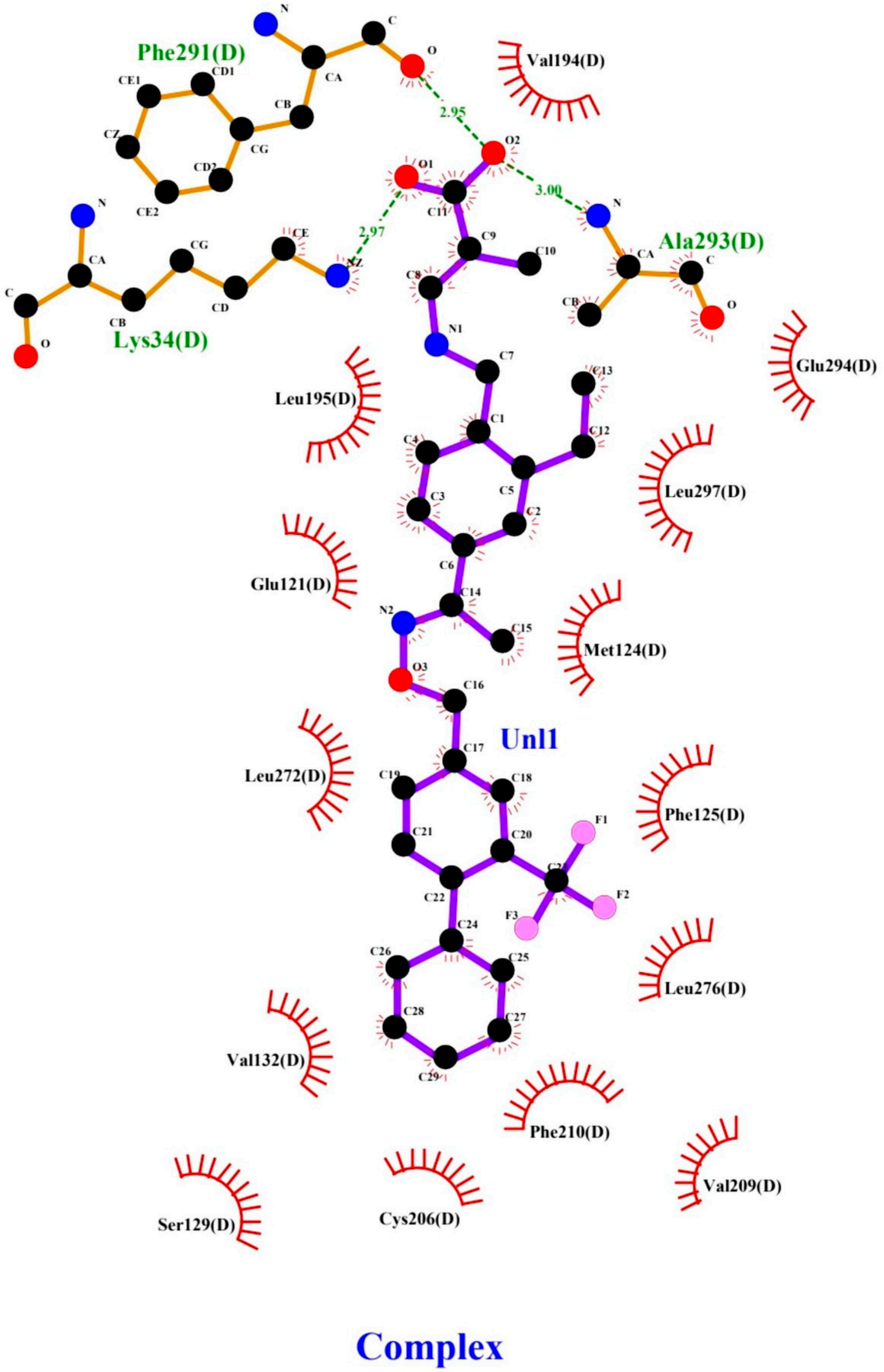

Figure S27. Docking interaction of DP-5 3D (**a**) and 2D (**b**) diagrams.

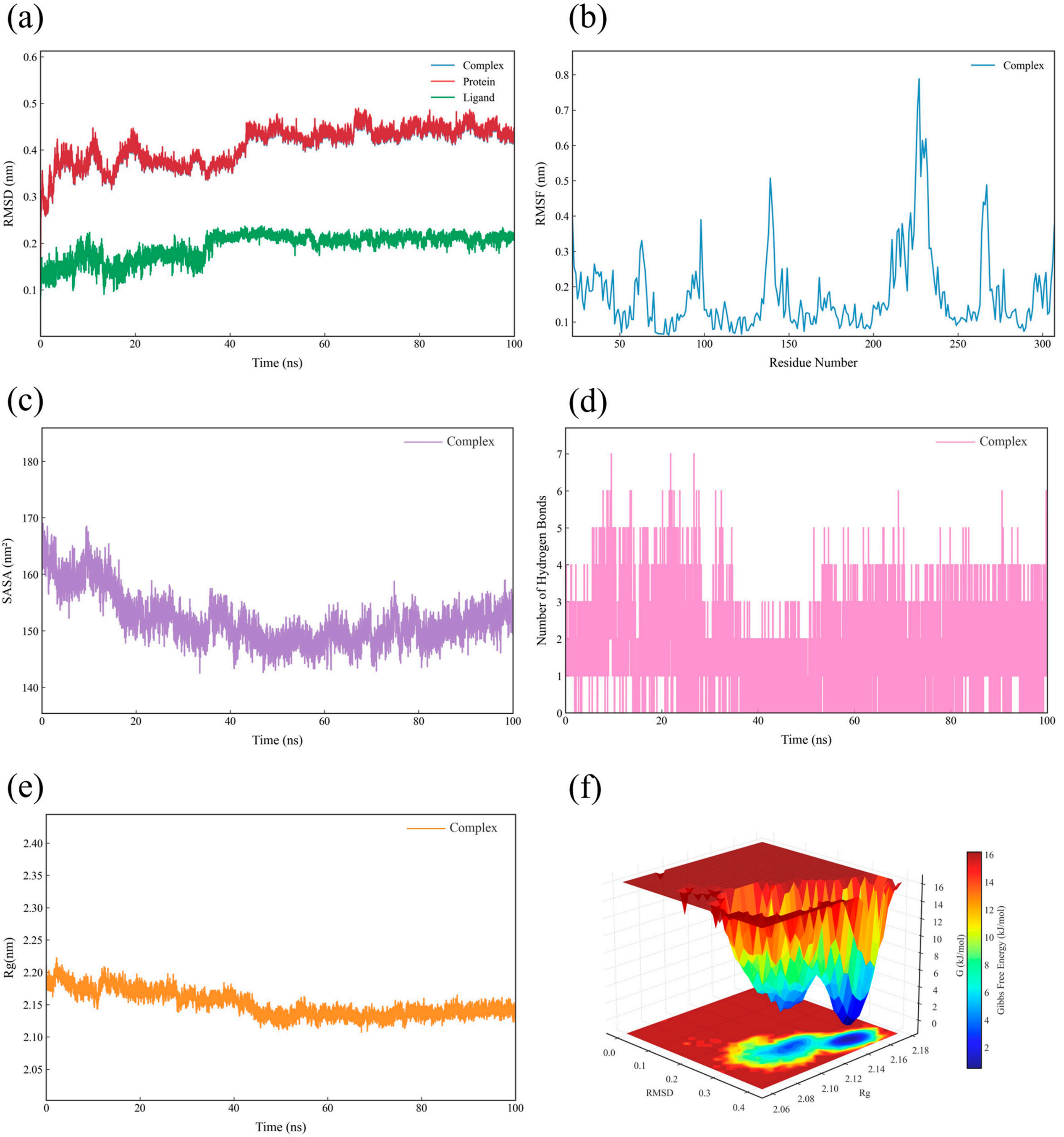

Figure S28. Examination of MD simulations data of siponimod: (**a**) RMSD of the complex; (**b**) Variation in RMSF of the complex; (**c**) SASA of the complex; (**d**) Count of hydrogen bonds within the complex; (**e**) Fluctuations in Rg of the complex; (**f**) Three-dimensional free energy profile.

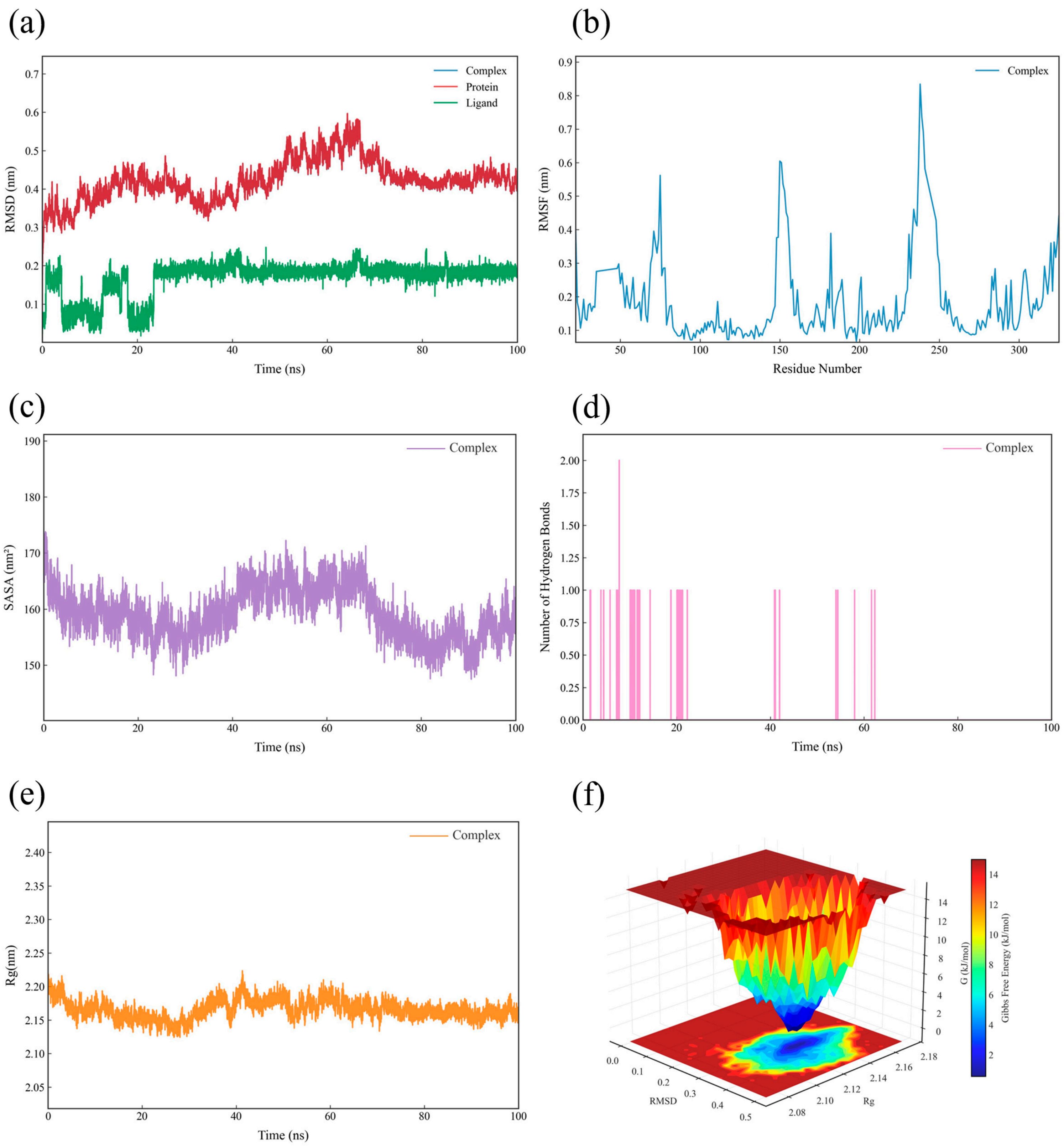

Figure S29. Examination of MD simulations data of DP-1: (**a**) RMSD of the complex; (**b**) Variation in RMSF of the complex; (**c**) SASA of the complex; (**d**) Count of hydrogen bonds within the complex; (**e**) Fluctuations in Rg of the complex; (**f**) Three-dimensional free energy profile.

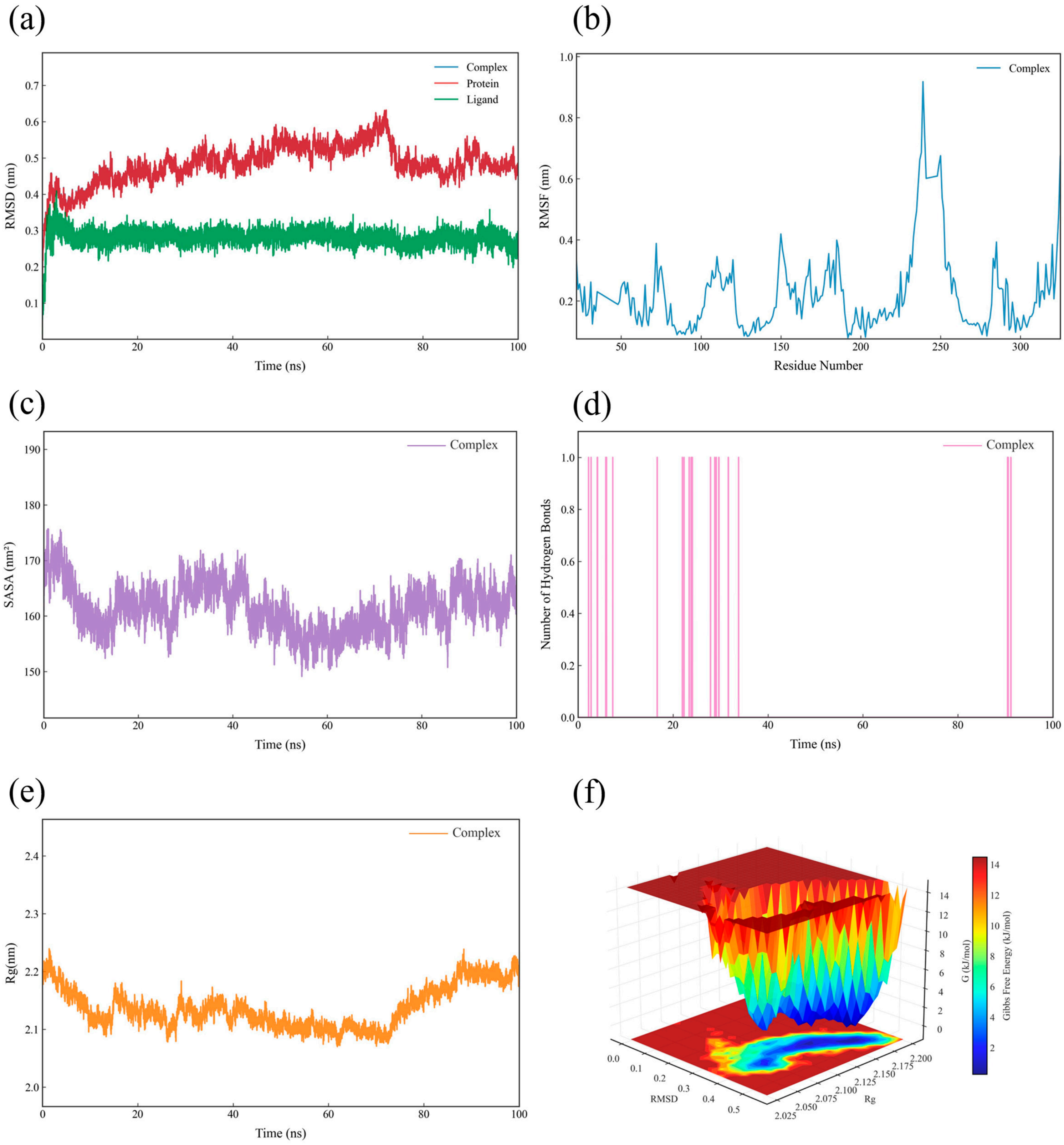

Figure S30. Examination of MD simulations data of DP-2: (**a**) RMSD of the complex; (**b**) Variation in RMSF of the complex; (**c**) SASA of the complex; (**d**) Count of hydrogen bonds within the complex; (**e**) Fluctuations in Rg of the complex; (**f**) Three-dimensional free energy profile.

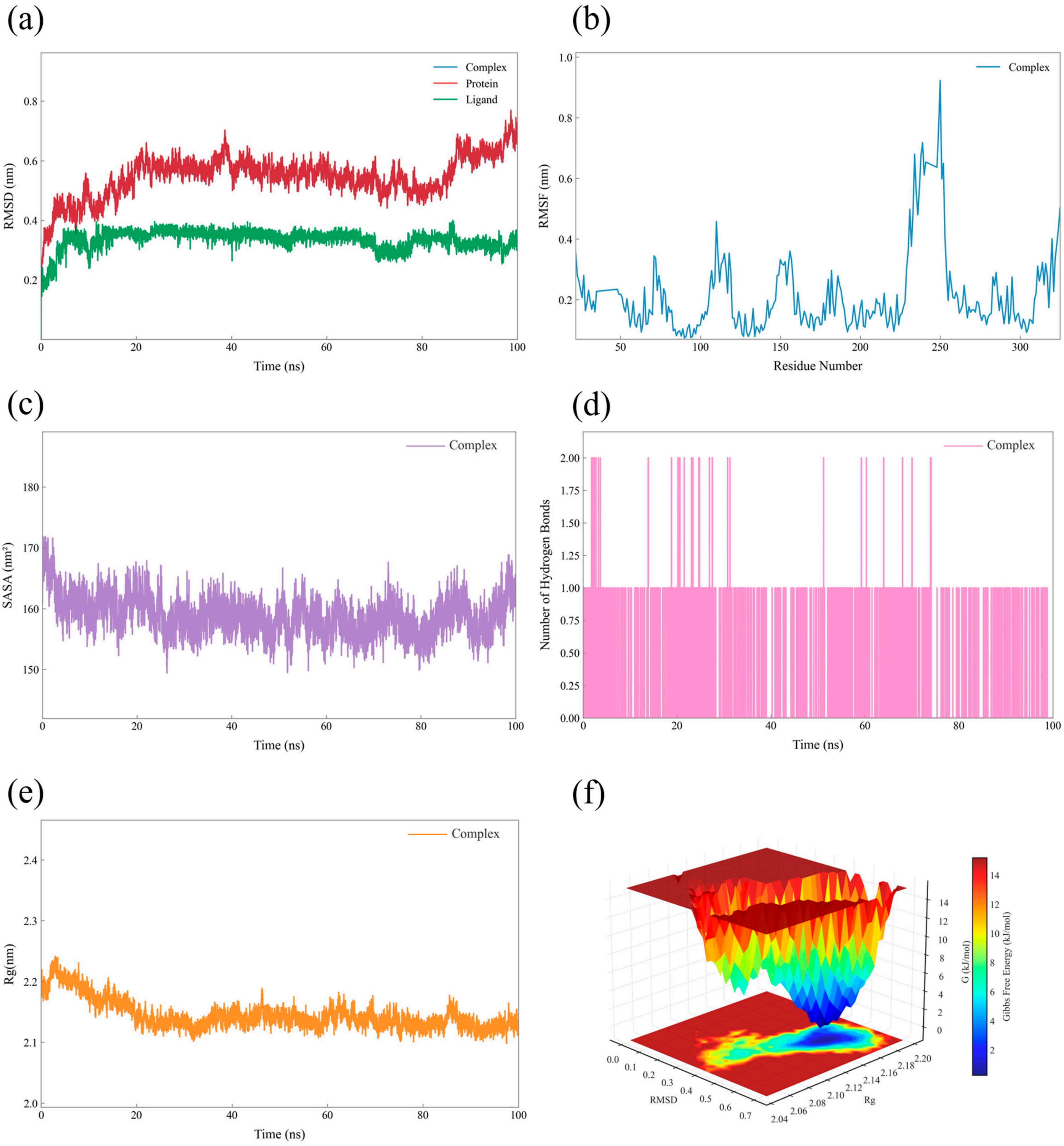

Figure S31. Examination of MD simulations data of DP-4: (**a**) RMSD of the complex; (**b**) Variation in RMSF of the complex; (**c**) SASA of the complex; (**d**) Count of hydrogen bonds within the complex; (**e**) Fluctuations in Rg of the complex; (**f**) Three-dimensional free energy profile.

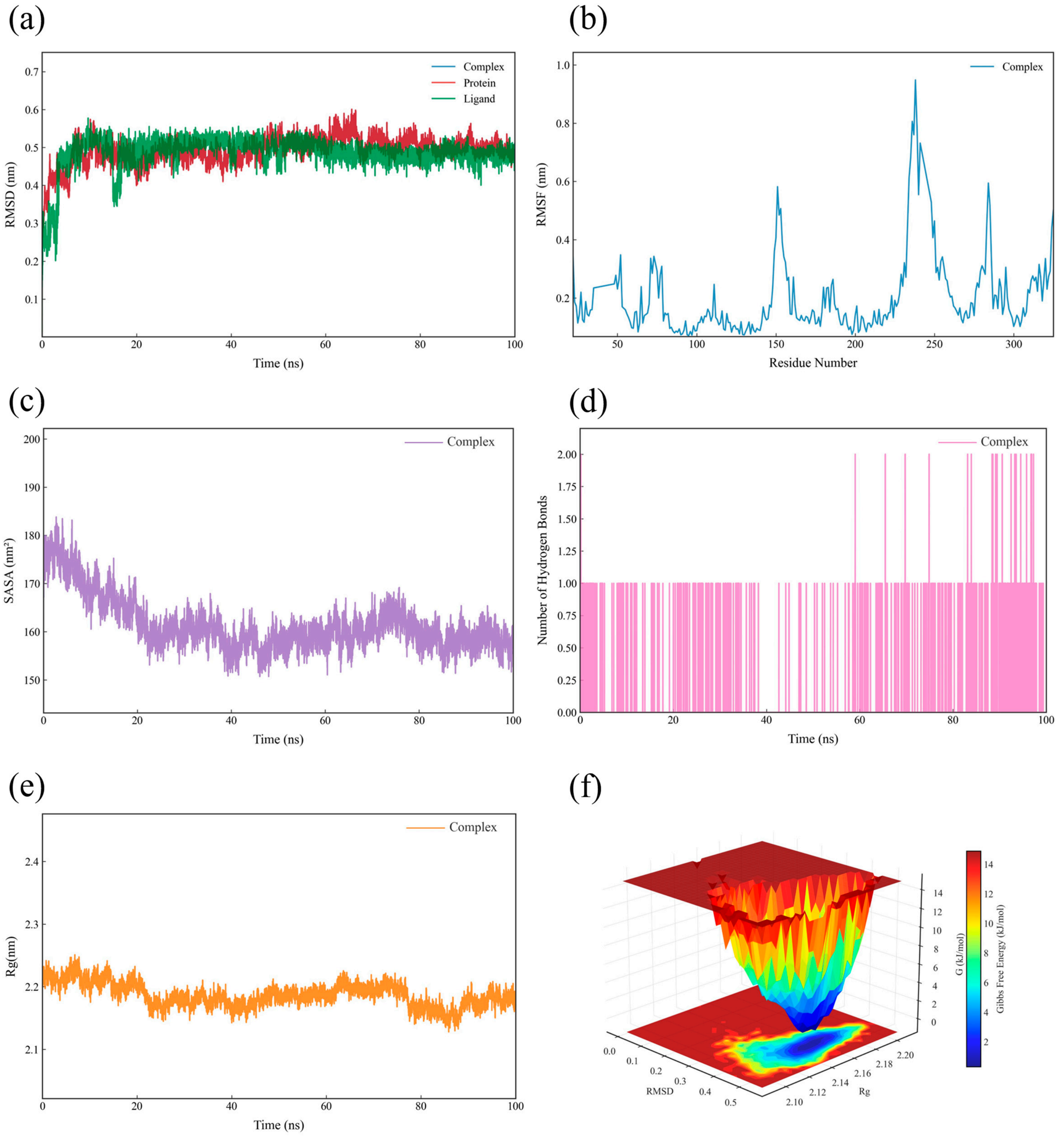

Figure S32. Examination of MD simulations data of DP-5: (**a**) RMSD of the complex; (**b**) Variation in RMSF of the complex; (**c**) SASA of the complex; (**d**) Count of hydrogen bonds within the complex; (**e**) Fluctuations in Rg of the complex; (**f**) Three-dimensional free energy profile.
